# Green synthesis of silver nanoparticles from *Mahonia fortunei* extracts and characterization of its inhibitory effect on Chinese cabbage soft rot pathogen

**DOI:** 10.3389/fmicb.2022.1030261

**Published:** 2022-10-21

**Authors:** Zhenlin Wei, Shuoqi Xu, Haoran Jia, Hongmei Zhang

**Affiliations:** Biological Sciences Department, Dezhou University, Dezhou, Shandong, China

**Keywords:** *Mahonia fortunei*, silver nanoparticles, antibacterial, cabbage soft rot, *Pectobacterium carotovorum*

## Abstract

The pathogenic bacterium *Pectobacterium carotovorum* causes soft rot in cabbage and significantly reduces plant yield. In this study, we employed *Mhonia fortunei* extracts to synthesis silver nanoparticles (Mf-AgNPs) and investigated their functions against *P. carotovorum*. The results showed that the surface plasmon resonance (SPR) peak of AgNP was 412 nm under optimal synthesis conditions. Furthermore, the results of Scanning electron microscope-Energy dispersive spectrometer (SEM-EDS) and High-resolution transmission electron microscopy (HR-TEM) revealed that the Mf-AgNPs had a spherical structure with an average diameter of 13.19 nm and the content of Ag0 ions accounted for 82.68% of the total elemental content. The X-Ray diffraction (XRD) results confirmed that AgNPs had a face-centered cubic (FCC) crystal structure, while Fourier transform infrared spectroscopy (FTIR) results indicated the presence of various biomolecules as reducing and stabilizing agents on the AgNP surface. Antibacterial activity was first evaluated by an inhibitory zone test, which revealed that 500 μg ml^−1^ of AgNPs had antibacterial activity against *P. carotovorum* and four model bacteria including *Staphylococcus aureus*, *Escherichia coli*, *Bacillus subtilis*, and *Pseudomonas aeruginosa*, respectively with an antibacterial function comparable to 1 mM AgNO_3_ solution. The Minimum inhibitory concentration (MIC) and minimum bactericidal concentration (MBC) values for *P. carotovorum* were 8 μg ml^−1^, respectively. Furthermore, AgNPs at 8 μg ml^−1^ completely inhibited the growth of *P. carotovorum*, decreased their tolerance to 0.25 mM H_2_O_2_ as well as considerably reduced colony formation after 1 h of treatment and thereafter. Treatment with Mf-AgNPs resulted in bacterial cell membrane destruction and biofilm formation inhibition, respectively. With an FIC (fractional inhibitory concentration) index of 0.174, AgNP and zhongshengmycin showed a significant synergistic effect. The infection of *P. carotovorum* to cabbage explants was significantly inhibited *in vitro* by a combination of 2 μg ml^−1^ Mf-AgNP and 5 μg ml^−1^ zhongshengmycin. In conclusion, the synthesized Mf-AgNP exhibited significant antibacterial activity against *P. carotovorum*.

## Introduction

In the last few decades, the creation and use of nanoparticles (NPs) have recently gained much interest and made great strides in their biological importance (Garg et al., 2020). The high surface area to volume ratio of the NPs causes them to have exceptional chemical, physical, and optical properties compared to their bulk structure (Susanti et al., 2022). Silver nanoparticles (AgNPs) are one of the most popular types of these NPs and have recently attracted much attention due to their high synthesis efficiency and significant applications, which boost their market value (Lee and Jun, 2019; Tarannum et al., 2019).

These days, various studies have demonstrated that AgNPs can combat a wide range of dangerous microorganisms, including bacteria, fungus, and viruses that affect food safety, agricultural yields, and human health (Bamal et al., 2021; Susanti et al., 2022). Due to its extremely deadly effects on bacterial cells, its immense potential as a bactericide has significantly expanded. Several methods of action have been established depending on their physiochemical features, such as charge, size, zeta potential, surface shape, and crystal structure, although the mechanism of AgNP cytotoxicity on bacterial cells has not yet been extensively investigated (Qing et al., 2018; Salleh et al., 2020; Susanti et al., 2022).

Numerous studies have discovered that AgNPs can injure cells *via* synergistic action of AgNPs and the release of Ag^+^ ions, referred to as contact killing and ion-mediated killing, respectively (Qing et al., 2018; Shaikh et al., 2019). In the case of the contact killing mechanism, the large surface area and positive charge properties allow the AgNPs to anchor to the bacterial cell wall through electrostatic attraction between the AgNPs and negative charge of the membrane of microorganisms, causing bacterial membrane rupture and subsequent cellular leakage of contents (Ibrahim et al., 2020). After entering the microbial cell, the AgNPs and the released Ag ions may interact with a variety of cell structures and macromolecules, leading to the enzyme malfunction and promoting the formation and accumulation of different types of reactive oxygen species (ROS), which in turn causes death (Qing et al., 2018).

Generally, AgNPs can be synthesized through multiple physical and chemical approaches, either “top to bottom” or “bottom to up” route (Lee and Jun, 2019). Although these methods are widely used in research, their main shortcomings for AgNPs synthesis are that they are pretty expensive or use hazardous substances as reducing and stabilizing agents, which are responsible for environmental problems and various biological risks (Garg et al., 2020).

As an alternative to these conventional techniques, a high-performance technique for the synthesis of AgNPs using components from plants and microbes, commonly known as the “green synthesis method,” has evolved (Vanlalveni et al., 2021). Among them, the manufacture of AgNPs *via* plant extractions has drawn much interest and use due to its affordable, easy for mass production, and environmentally benign (Lee and Jun, 2019; Vanlalveni et al., 2021). The use of plant extracts in the “top to bottom” process of AgNP production depends on the multiplicity of biomolecules operating cooperatively as reducing and stabilizing agents.

Depending on the plants employed, different natural ingredients, such as proteins, amino acids, enzymes, polysaccharides, alkaloids, tannins, phenols, saponins, terpenoids, and vitamins, were used to produce AgNPs (Shaikh et al., 2021). Among the plants used for green synthesis of AgNPs, natural medicinal plants have been extensively applied, due to their benefits of broad sources and diverse active ingredients. The alkaloids, polysaccharides, and flavonoids contained in these plants have antibacterial activities and are also reducing agents and stabilizers in the synthesis of AgNP (Salayová et al., 2021; Habeeb et al., 2022). Furthermore, the capped surface with the active ingredients enhances the antibacterial ability of AgNP (Wei et al., 2019; Salayová et al., 2021; Habeeb et al., 2022).

Chinese cabbage (*Brassica rapa* ssp. pekinensis) is one of the most important vegetables in Eastern Asia (Liu et al., 2019). However, the disease of cabbage soft rot has recently emerged as one of the three top diseases in cabbage cultivation worldwide and has become the most severe and destructive disease factor limiting the development of cabbage (Cui et al., 2019; Liu et al., 2019). Regarding the etiology, soft rot of cabbage is a bacterial disease mainly caused by the bacterium *Pectobacterium carotovorum* subsp.*carrot* in the genus of *Pectobacterium*, which is formerly known as *Erwinia carotovora*. Additionally, *P. carotovorum* is a common pathogenic bacterium causing soft rot in various vegetable hosts, including Chinese cabbage, tomato, potato, and cucumber, resulting in loss of marketable yield (Cui et al., 2019).

Traditional chemical drugs are not desirable due to concerns regarding the development of resistance, environmental pollution, and high cost (Cai et al., 2022). Therefore, more effective and environmentally friendly methods for controlling this disease are required to reduce the use of chemical pesticides (Cui et al., 2019).

The plant *Mahonia fortunei* is a typical Chinese medicine with a special antibacterial function. Its main active ingredients include polyacetylenes, terpenoids, diterpenes, triterpenes, and triterpene saponins, phenols and flavonoids, which have antibacterial and anti-tumor effects (Li et al., 2007; Yuan et al., 2017b). However, the synthesis of AgNP using the extract of *M. fortunei* and the characterization of its bactericidal activity on *P. carotovorum* still lack sufficient research. In this research, we first synthesized AgNPs using the extraction of *M. fortunei*, and determined their bacteriostasis activities solely and in combination with Zhongshengmycin. The results demonstrate that the bio-synthesized AgNPs can effectively control *P. carotovorum.* This function was more remarkable in combination with Zhongshengmycin, demonstrating its application for the further development of highly efficient antibacterial ingredients at low cost.

## Materials and methods

### Materials

Silver nitrate (AgNO_3_) was purchased from Sigma-Aldrich (Sigma-209,139). All other reagents used in this research were obtained from Sangon Biotech (Shanghai) Co., Ltd. of analytical grade. The whole plant of *M. fortunei* was obtained from the market of Guangxi province, China.

Four bacterial strains, including *S. aureus* (ATCC 25923), *E. coli* (ATCC 1399), *B. subtilis,* and *P. aeruginosa* (ATCC 1430) were preserved in our laboratory and used for antibacterial studies. The strain of *P. carotovorum* was provided by Prof. Yueqiu He of Yunnan Agricultural University. Meanwhile, the human pancreatic cancer cell line CFPAC1 and a human skin fibroblast cell line HFS were supplied by Dr. Xiangyong Li.

### Bio-synthesis of Mf-AgNPs

The whole plant was thoroughly rinsed with sterilized deionized water and dried for 24 h in a vacuum oven at 65°C. Then, the dried plant (10 g) was finely ground using a pulverizer and was transferred into a 250 ml Erlenmeyer flask containing 100 ml Milli-Q water and boiled for 20 min. After the time, the extract was filtered through Whatman number 1 filter paper to obtain the pure extract (Rajivgandhi et al., 2020a). The total phenolic content (TPC) and total flavonoid content (TFC) were determined according to Rajivgandhi et al. (2019a, 2021).

As both the reducing agent and stabilizer during the reaction, the extract solution was mixed with 1 or 2 mM AgNO_3_ solution for AgNP synthesis with vigorous stirring at 37°C. The green synthesized Mf-AgNPs were successfully produced by the visual appearance of a typical dark brown color (Rajivgandhi et al., 2022a).

In this step, three main factors affecting the particle size of synthesized Mf-AgNP were further investigated, including reactant concentration of extract, reaction time, and pH of extraction (Ndikau et al., 2017; Shaikh et al., 2021). Briefly, the pH value of the extract solution was adjusted to 7.0, 8.0, 9.0, 10.0, and 11.0 using 0.1 M NaOH solution, followed by adding 10 ml extracts into 90 ml of 1 mM AgNO_3_ solution and agitating on a magnetic stirrer at 37°C for 1 h.

Second, 10 or 20 ml of the extract was mixed with 90 or 80 ml of 1 mM AgNO_3_ solution to obtain the optimal ratio of extract and AgNO_3_ solution. Third, the reactions prepared with the optimized extract pH and the ratio of two reactants were continued for 1, 2, 4, and 6 h to find the best reaction duration. The parameter’s optimization was evaluated by using the Uv–Vis absorbance spectroscopy analysis on a spectrophotometer (Evolution 220, Thermo Fisher; Aritonang et al., 2019).

After synthesis, the AgNPs were precipitated by centrifugation at 12,500 rpm for 30 min at ambient temperature and washed twice with Milli-Q water to remove unreacted substances. The black precipitate was lyophilized, suspended with Milli-Q water at a concentration of 1 mg ml^−1^ and stored at 4°C for further research.

### Characterization of synthesized AgNPs

In this study, the Mf-AgNPs were characterized by various physical and chemical methods to elucidate their optical properties, structure, composition, and surface morphology. First, the surface morphology and elemental composition of AgNPs were confirmed by SEM (Merline compact, Zeiss, Germany) coupled with EDS (Rajivgandhi et al., 2020b). The shape and particle size distribution of MfAgNPs were recorded using HR-TEM (FEI, Talos F200X G2) and estimated using Nano Measurer 1.2 software (Yuan et al., 2017a; Rajivgandhi et al., 2019a).

The XRDcrystalline pattern of Mf-AgNPs was recorded on a Panalytical Emperian diffractometer (Ultima 4, Japan) with a step size of 0.02° and a scan speed of 5°/min in the scan range from 30° to 80° of 2 (Rajivgandhi et al., 2020a). Using the width of the peak with the most pronounced Bragg’s reflection as a reference, Debye-equation Scherrer’s was used to determine the average crystalline size of the AgNPs (Sharma et al., 2018).

The FTIR (Fourier Transform infrared spectroscopy) analysis was performed to identify functional groups responsible for reducing, stabilizing, and capping of Mf-AgNPs. The FTIR spectra were collected on Nicolet (iS50, Waltham, MA, United States) by scanning a range of 4,000–400 cm^−1^ with a resolution of 1 cm^−1^ (Hussain et al., 2019). Lastly, the average size (hydrodynamic volume) of the Mf-AgNP was determined using the dynamic light scattering (DLS) method on a Zetasizer Nano ZS device (Malvern, England; Yuan et al., 2017a), and the Zeta potential of Mf-AgNPs were obtained simultaneously.

### Determination of the antibacterial activity of Mf-AgNPs

#### Agar diffusion assay

First, the AgNPs were tested for antimicrobial activity by a standard agar diffusion assay against five bacterial cultures, *S. aureus*, *E. coli*, *B. subtilis P. aeruginosa,* and *P. carotovorum* (Aritonang et al., 2019). Before testing, each bacterial strain was inoculated overnight and adjusted to the colony-forming unit (CFU) of 10^7^ per ml with fresh Luria-Bertani medium (LB medium, pH 7.0).

For each strain, 100 μl of bacterial culture was spread on the solid LB medium, and five Oxford cups were placed thereon. Then, 100 μl of AgNPs solution (500, 250 and 125 μg ml^−1^) was added into the oxford cup, with an equal volume of 1 mM AgNO_3_ and the extract solution was added as negative and positive controls, respectively. These LB solid mediums were then incubated at 37°C or 28°C for 12 h. The inhibition zones were measured and were used to evaluate the antibacterial activities (Qais et al., 2019).

#### Minimum inhibitory concentration and minimum bactericidal concentration determination

The minimum inhibitory concentration (MIC) assay was determined on 96-well microtiter plate. In this research, 100 μl of each tested bacterial culture was added to the wells, followed by the addition of Mf-AgNPs solutions to the final concentration from 512 to 0.25 μg ml^−1^ in the manner of twofold gradient dilution, respectively. The wells mixed with 100 μl liquid LB medium alone or 100 μl bacterial cultures were designated as the negative or positive control, respectively. After 16 h, 10 μl of 2,3,5-triphenyl tetrazolium chloride solution (TTC, 0.5%) was added to each well as a color indicator of bacterial growth (Rajivgandhi et al., 2021).

The MBC (minimum bactericidal concentration) values of AgNPs against four microorganisms were determined on solid LB plates with the same Mf-AgNPs concentrations as in the MIC assay (Loo et al., 2018).

#### Growth curve experiment

The optical density (OD) measurements at 600 nm were used to evaluate the effect of Mf-AgNPs on the growth of *P. carotovorum*. Bacteria were grown overnight and diluted 1/100 fold in the presence of Mf-AgNPs at a final concentration of 0.5 MIC, 1 MIC, and 2 MIC, respectively. The bacterial culture was grown at 28°C conditions, and the OD value was recorded over 12 h at 2 h intervals (Qais et al., 2019).

#### Time-killing assay

With some modifications, the time-killing assay was performed as described by Loo et al. (2018). The bacterial inoculums were adjusted to 10^7^ CFU mL^−1^ and were challenged by AgNPs (0 × MIC, 0.25 × MIC, 0.5 × MIC, and 1 × MIC) for 2 h. The cultures were then incubated at 28°C with 150 rpm agitation for 0, 0.25, 0.5, 1, 2, and 4 h, respectively. On completion, the cultures (100 μl) were spread on LB plates, and the number of colonies on plates was quantified in Log_clone numbers_ after incubation at 28°C for 16 h (Loo et al., 2018).

#### Bacterial cell membrane leakage

We performed OD measurement and SEM observation methods to detect cell membrane leakage induced by Mf-AgNP. In the OD measurement assay, 2 ml of bacterial culture (1 × 10^7^ CFU mL^−1^) were harvested by centrifugation at 4,000 rpm and washed twice with PBS (pH7.4), treated with AgNP in PBS to a final concentration at 0.5 × MIC, 1 × MIC, and 2 × MIC. Then, the supernatant was obtained after 0.5–4 h by centrifugation at 5,000*g* for 10 min and was subjected to OD measurement at 280 and 260 nm, respectively. The bacterial cells without AgNP treatment were controls (Ibrahim et al., 2020).

Meanwhile, the *P. carotovorum* cells were treated with Mf-AgNP (1 × MIC) for 0–2 h, then the cells were centrifuged at 4,000 rpm for 10 min and the pellets were washed twice with PBS (pH7.4). The harvested bacterial cells were resuspended in PBS and fixed with 2.5% glutaraldehyde for 3 h. Subsequently, the bacterial cells were subjected to gradient dehydration with 30%–100% ethanol, each for 15 min. Finally, the surface morphology of the bacteria was observed using SEM (Muthuchamy et al., 2019).

#### Hydrogen peroxide tolerance assay

For device limitation, we used the hydrogen peroxide tolerance method according to Lan et al. (2010), to assess the redox status of *P. carotovorum* under different AgNP treatments. Briefly, the bacterial culture (1 × 10^7^ CFU mL^−1^) was treated with AgNP (1 × MIC) for 0.25, 0.5, 1, and 2 h, then the cells were diluted four times in a tenfold gradient, and 2 μl of each dilution was seeded onto the plates containing H_2_O_2_ at concentrations of 0.1 mM, 0.25 mM, and 0.5 mM, respectively. Next, the plates were incubated in an incubator at 28°C for 16 h. Finally, the tolerance of each sample to hydrogen peroxide was evaluated by observing the colony formation.

#### Determination of extracellular pectinase and cellulase activities

The enzymatic activities determination was performed according to Chen’s (2008) method. To minimize the inhibitory effect of Mf-AgNP on the growth of *P. carotovorum,* which would reduce the density of bacterial test cells, we used very low concentration AgNP (1 μg ml^−1^) to treat the bacterial culture (1 × 10^7^ CFU ml^−1^) for 2 h, to determine the direct effect of AgNP on the activity of these two types of enzymes. After challenge, 2 μl of each bacterial culture tested was inoculated onto the pectinase assay plate (yeast extract 0.1%, (NH_4_)_2_SO_4_ 0.1%, glycerol 0.5%, MgSO_4_ 1 mM, sodium polygalacturonate 0.5%, PBS 20%, agar 1.6%, pH 8.0) and was incubated at 28°C for 24 h. The pectinase plate was then stained with 2 ml of 7.5% copper acetate staining solution for 1 h before being washed twice with water. The size of the transparent circle around the colony against the blue–green background of the plate was used to compare the difference in pectinase activity between strains.

At the same time, the activity of cellulase was determined using other type of plates (carboxymethyl cellulose 0.1%, yeast extract 0.5%; (NH_4_)_2_SO_4_ 0.1%, glycerol 0.5%, MgSO_4_ 1 mM, sodium polygalacturonate 0.5%, agar 1.6%, all agents were dissolved in PBS buffer, pH 7.0). These plates were processed the same as the pectinase activity assay, but strained with 0.1% Congo red staining solution for 20 min and washed twice with water and 1 mM NaCl, respectively.

#### Anti-biofilm activity

The crystal violet staining experiment was applied to investigate the anti-biofilm activity of Mf-AgNPs. To begin with, *P. carotovorum* (1 × 10^8^ CFU mL^−1^) was cultured on a 96-well plate for 36 h to allow biofilm formation. After the time, the methanol-fixed biofilm was stained with crystal violet (0.1%, dissolved with acetic acid), resolved with 33% acetic acid, and the OD_570_ absorbance was recorded with a microplate reader. The biofilm destruction rates of AgNP were calculated according to the following formula: Destruction Rate % = (OD_570 Control_ − OD_570 Sample_)/OD_570 Control_ (Habibipour et al., 2019).

### Synergistic inhibition of AgNP with Zhongshengmycin

Briefly, the MIC concentration of Zhongshengmycin was first tested using a 96-well plate. Then the synergistic effect of AgNP with zhongshengmycin was performed using the checkerboard method, and the concentration range of zhongshengmycin was 200, 100, 50, 25, 12.5, 6.25 and 3.1 μg ml^−1^, with seven gradients, while the concentration of AgNP was set to 200, 100, 50, 25, 12.5, 6.25, 3.12, 1.6, 0.8, 0.4, 0.2 and 0.1 μg ml^−1^, with 12 gradient sets. After 16 h, 10 μl of 0.5% TTC solution was added to each well to clarify the growth of bacterial cells. Hence, the FIC index (fractional inhibitory concentration) was calculated using the formula (MIC of AgNPs in synergy with zhongshengmycin/MIC of AgNPs alone) + (MIC of zhongshengmycin in synergy with AgNPs/MIC of zhongshengmycin alone; Wypij et al., 2018).

### *In vitro* counter *Pectobacterium carotovorum* effect of AgNP and Zhongshengmycin

The petioles near the base of Chinese cabbage were first disinfected with a 75% alcohol wipe, cut into ~4 cm stem segments with a sterilized scalpel, put onto a sterile filter paper underneath, and placed in a sterile Petri dish. Subsequently, 2 ml of sterile distilled water was added to the filter paper to maintain humidity. Next, a cruciform wound of 2 mm × 2 mm was cut at the center of each explant with a sterilized scalpel at a depth of about 1.5 mm, and 10 μl of *P. carotovorum* bacterial suspension of 1 × 10^7^ CFU ml^−1^ treated with different concentrations of AgNP and zhongshengmycin was added into the wound. After incubating at 28°C for 12 h, the lesions were observed and compared with controls (Qiao et al., 2018).

### *In vitro* cytotoxicity assay

The cytotoxicity of Mf-AgNPs was evaluated using the MTT (3-(4,5-dimethylthiazol-2-yl)-2,5-diphenyltetrazolium bromide) assay by measuring the metabolic activity of the cells (Hussain et al., 2019). The two cell lines (20,000 cells/well) were cultured in DMEM supplemented with 10% fetal bovine serum (FBS) at 37°C in a 5% CO_2_ incubator. After 24 h of culture, the cells were treated with different dosages of AgNPs (0.39–100 μg ml^−1^) in the growth medium for another 24 h under the same culture conditions, followed by adding 100 μl of MTT (0.1 mg ml^−1^) to the respective wells and incubating for 4 h in darkness. Then, the contents of formazan that dissolved in isopropanol, were quantified by measuring absorbance at 570 nm. The percentage of cell proliferation was calculated by taking (absorbance of the sample)/(absorbance of control).

### Statistical analysis

The data were given as mean ± SE after replications of each experiment. Analysis of variance (ANOVA) was utilized to determine the significant difference (*p* < 0.05) between the tested samples for statistical analysis using the statistical program Graphpad Prism (v.8.3.0). Using Tukey’s test, significant differences between the means were determined (Rajput et al., 2020).

## Results

### Green synthesis of Mf-AgNP

**Figure 1:**
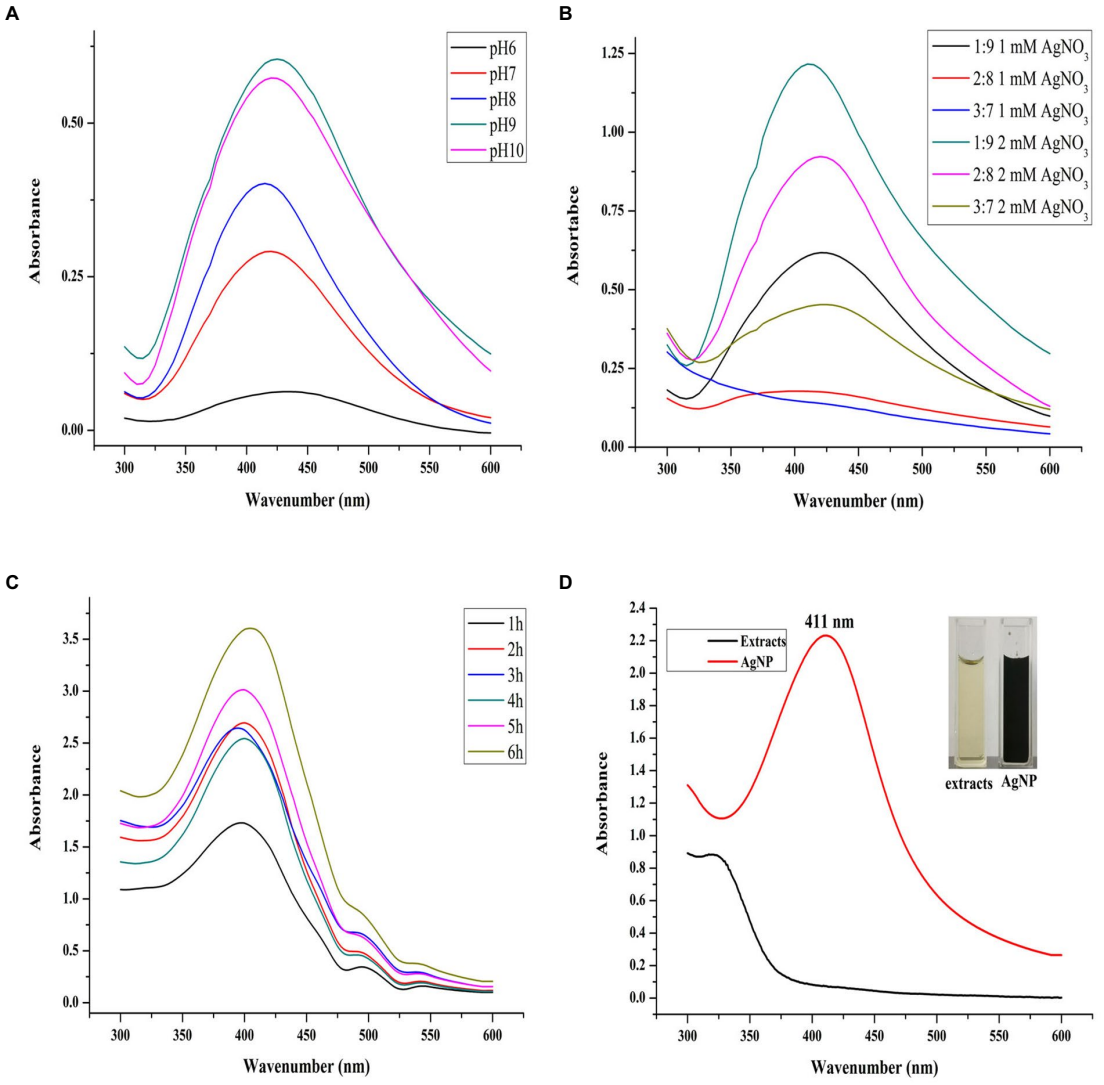
pH value, synthesis ratio, and synthesis time on AgNP synthesis. The panels **(A–C)** refer to the Uv–Vis scanning spectra of AgNPs synthesized at different pH values, extract to AgNO_3_ ratios, and reaction times, respectively. The panel **(D)** refers to the synthesis of AgNPs at the optimal parameters and their color change.

In this study, the Uv–Vis spectral scanning was used to identify of successful formation of Mf-AgNP and to optimize the synthesis parameters, since the AgNP exhibits the surface plasmon resonance (SPR) peak in a range from 400 to 450 nm (Ndikau et al., 2017; Sytu and Camacho, 2018). First, we optimized the three primary parameters affecting AgNP biosynthesis. Among them, the optimal pH had to be determined first since it was the most critical factor in AgNP biosynthesis (Darroudi et al., 2010). As shown in Figure 1A, the pH of the reaction solution has a profound impact on the process of AgNP biosynthesis. At pH 6, AgNP can hardly be synthesized. In contrast, the yield of AgNP increased sharply upon increasing the pH of reactants from 7 to 10, as evidenced by the appearance of the intense SPR peak of AgNPs in Uv–Vis spectrum. Furthermore, higher pH accelerated AgNP formation, resulting in a faster color change of the reaction mixture. The extract solution without AgNO_3_ on the other hand showed no color change and no SPR peak in its Uv–Vis spectrum.

This phenomenon has been observed in numerous studies as a result of the activation of a group of phenolic-based plant reductive substances with increased pH, allowing for the synthesis of small particle-size AgNP (Ndikau et al., 2017). Although under pH 9, the AgNP production was most significant, its SPR peak location moved to a higher wavenumber than that of AgNPs synthesized under pH 8, shifting from 414 to 425 nm. It has been found that the smaller SPR position indicates the particle size of AgNP changes to a smaller state. The result indicated that pH 8 was the optimal condition for AgNP synthesis and was selected during the subsequent study. Previous studies have shown that alkaline conditions are favorable for the synthesis of Mf-AgNPs (Shaikh et al., 2021; Vanlalveni et al., 2021) and similar results were obtained in this study.

Next, the optimal ratio of reactants (by volume) was evaluated. Under the combination of three levels of plant extracts and two AgNO_3_ concentrations, the AgNP synthesis results showed that under the ratio of the substances to 2 mM AgNO_3_ is 1:9 (Figure 1B), the Mf-AgNP yield was the maximum. In addition, the sharpness of the SPR peak was also increased and its localization was shifted to a lower wavenumber, suggesting a more concentrated distribution of particle size of Mf-AgNP and smaller particle size. As a result, this volume ratio was chosen as one of the best parameters for the following synthesis.

The reaction time has a significant impact on the synthesis of Mf-AgNP (Figure 1C), and the results revealed that a reaction period of 6 h produced the largest amount of AgNP and that their SPR peak positions did not significantly differ from one another. This study discovered that the ideal AgNP synthesis reaction time was longer than that of many others, which commonly ranged from 30 min to 3 h (Rajput et al., 2020). The fact that the yield of bio-synthesized Mf-AgNP increases proportionally with temperature led us to infer that this result is mostly due to the lower reaction temperature at 37°C instead of 60°C, which prolonged the synthesis period (Mat Yusuf et al., 2020; Vanlalveni et al., 2021). Overall, the optimal synthesis conditions were: ratio 1:9, pH 8, and 6 h, generating a narrow SPR peak at 412 nm (Figure 1D). These results suggested a smaller size AgNPs were obtained and used for antibacterial evaluation since the particle size of AgNPs has a decisive influence on their antimicrobial activity (Qing et al., 2018).

### Characterization of AgNPs

**Figure 2:**
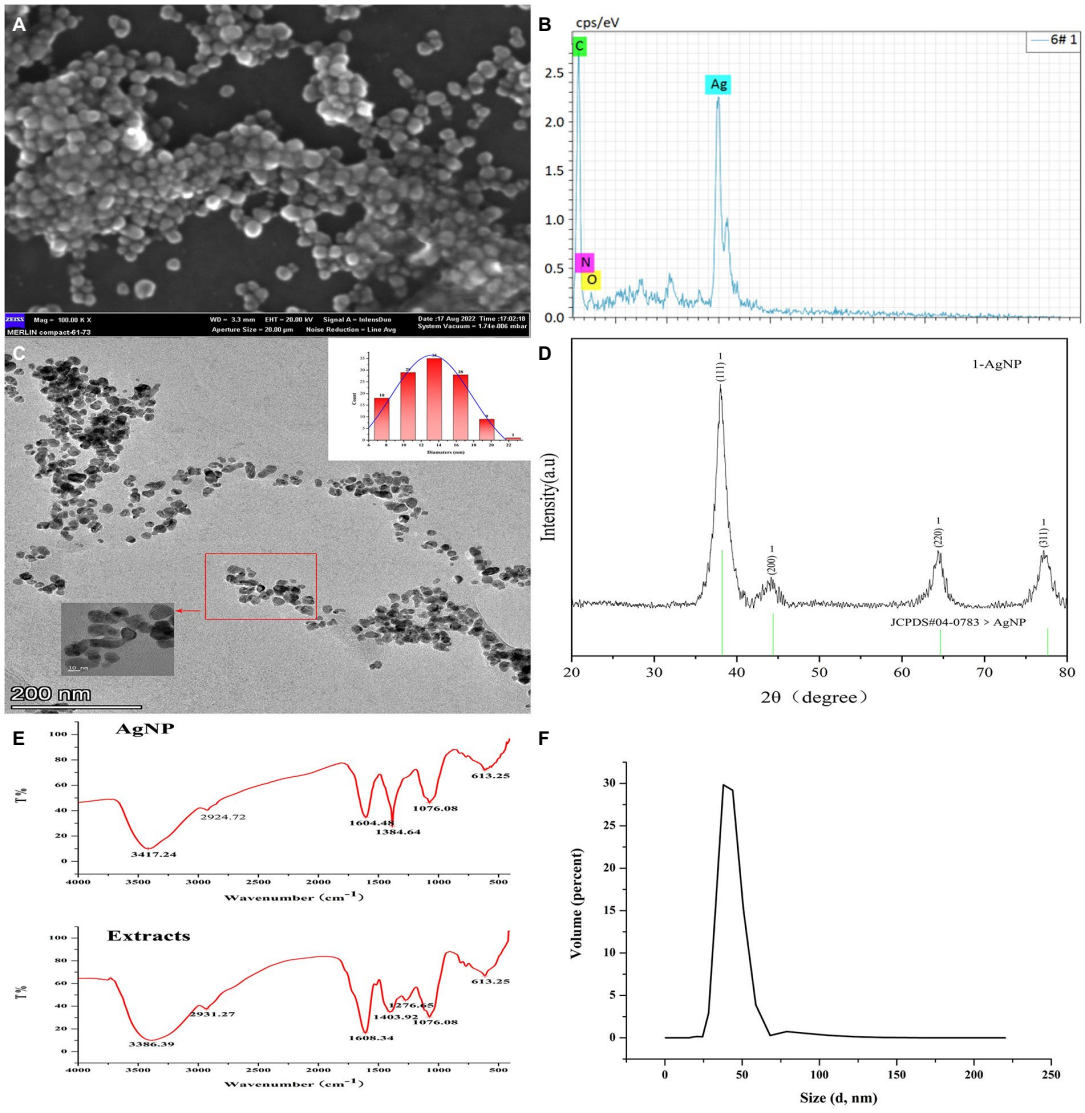
Characterization of Mf-AgNPs. **(A)** SEM image of AgNPs; **(B)** EDS result; **(C)** HR-TEM image and particle size distribution of AgNPs; **(D)** XRD results; **(E)** FTIR spectrum of AgNPs; **(F)** DLS measure result.

Initially, the surface morphology and elemental compositions of the synthesized AgNPs were determined by SEM scanning equipped with an EDS detector. It showed approximately spherical Mf-AgNPs in clusters due to the aggregation of the smaller particles after high-speed centrifuging during sample preparation (Figure 2A). The slight agglomeration of metal based nano-materials have been observed in previopus researches (Rajivgandhi et al., 2021, 2022a). An additional EDS spectrum displays the elemental compositions of the Mf-AgNPs with the presence of two characterized silver peaks at 3 and 3.1 Kev, originating from silver L and K shells, respectively (Zada et al., 2018). The silver peak was the most intense among all the peaks, accounting for 85% weight percentage of the total elements, as a signature of Ag is the major element in AgNPs. In addition, the C element accounted for 14.34%, then followed by the O and N elements (Rajivgandhi et al., 2020b). The presence of peaks corresponding to the C, N, and O elements confirmed that metabolites from *M. fortunei* served as capping molecules (Mat Yusuf et al., 2020; Figure 2B).

Next, the HR-TEM and DLS determination was used to obtain the finer details regarding the size and morphology nature of Mf-AgNPs (Figures 2C,F). As shown, the as-synthesized Mf-AgNPs were monodispersed, spherical in shape with smooth edges. Their particles ranged in diameter from 6.8 nm to 21.2 nm with an average of 13.19 ± 3.4 nm, mainly concentrated at 14 nm. The results were comparable to other research (Vanlalveni et al., 2021). The mean particle size of Mf-AgNPs fell into the appropriate size range (8–50 nm) for biological membrane permeation, and this is the tolerable range for inducing toxicity within cells (Samuggam et al., 2021). Notably, a few agglomerated AgNPs were also observed in some places, which was consistent with the results of the zeta-potential determination. Therefore the synthesis conditions need additional optimization to obtain more consistent Mf-AgNPs. Furthermore, the DLS measurement revealed that the hydrodynamic diameter was ranging from 28 to 68 nm for Mf-AgNP with two dominating peaks at 38.14 and 43.82 nm (Figure 2F), respectively. Meanwhile, the zeta-potential was −11.5 mV (Supplementary Figure S2), which ensured the Mf-AgNP moderate stability.

The FTIR analysis was used to characterize the functional groups on the surface of AgNP and in plant extracts, and the results revealed that the spectra of these two samples were similar, indicating that the capping substances of AgNP originated from the extracts. As shown in Figure 2E, seven intense peaks at 3386, 2931, 1,608, 1,403, 1,276, 1,076, and 613 cm^−1^ were identified in extracts, while the same number of peaks were identified with slight shifting (Supplementary Table S1). Among them, the broad and intense bands at 3386 and 3,417 cm^−1^ can be assigned to the stretching vibration of –OH (hydroxyl groups) of phenols and carboxylic acids, while the weak peaks around 2,931 and 2,924 cm^−1^ both were asymmetric stretching of the C–H bonds of aliphatic acids or methylene groups (−CH_2_) in aliphatic hydrocarbons, respectively (Ashour et al., 2015; Yang et al., 2021). The peaks around 1,600 cm^−1^ signified the amide I (–N–H) group of peptide bonds. The peaks at 1,384 and 1,401 cm^−1^ indicated the O–C–H and C–O–H bending vibrational modes (in-plane) of the carbohydrates or hydroxyl flavones (Ibrahim et al., 2020; Mat Yusuf et al., 2020). The peak around 1,076 represented the C-O (carboxylic acid) stretching vibrations of aromatic compounds, while (Saratale et al., 2018) the broad peak around 613 cm^−1^ might be due to the C-H bonding of aromatic compounds (Reddy et al., 2021). These results suggested the presence of flavanones or terpenoids on the surface of Mf-AgNP (Logaranjan et al., 2016). The results of FTIR spectra clearly indicated the involvement of different functional groups of phytochemical constituents of extracts serving as the reduction and capping components in the Mf-AgNPs synthesis, especially plant phenols and flavonoids. Well agree with this result, the quantitative determination of TPC and TFC showed that the extract from *M. fortunei* was rich in plant phenols and flavonoids, which reached 352.54 ± 10.74 and 241.37 ± 10.729 mg L^−1^, respectively (Supplementary Figure S1).

The crystalline nature and the presence of the Ag^0^ element on the surface of the Mf-AgNPs were determined based on the XRD analysis and the result agreed well with that of previous studies (Vijayan et al., 2018; Yang et al., 2021). The results showed that there were four emission peaks of 2*θ* = 38.00, 44.16, 64.34, and 77.18, corresponding to the face-centered cubic (FCC) silver crystal planes (111), (200), (220), and (311) of the Joint Committee on Powder Diffraction Standard (JCPDS, Card No.04–0783), respectively (Figure 2D). These results confirmed that the nanoparticles were composed predominantly of elemental silver. In addition, it clearly showed that the peak related to the (111) plane is the most intense among the XRD peaks, thereafter suggesting that AgNPs formed in this present synthesis are crystalline in nature with FCC structure (Vijayan et al., 2018). Furthermore, the appearance of an intense peak at 2*θ* = 38° and two weak peaks at 2*θ* = 44° and 64° indicated the Ag^0^ element on the surface of Mf-AgNPs (Yang et al., 2021).

The peaks raised by other Ag compounds such as AgCl and Ag_2_O were absent from this pattern, indicating the extract of *M. fortunei* has sufficient reducing power to form pure metallic AgNP (Okaiyeto et al., 2019; Yang et al., 2021). Another reason contributing to the complete reduction of Ag^2+^ to Ag^0^ was the pH 8 condition used in this study. This pH was not a hash pH parameter and was able to avoid the formation of AgOH by-product (Vanlalveni et al., 2021), thus exhibiting a pure Ag^0^ spectral peak.

In addition, the positions of the peaks were slightly shifted due to the presence of strains in the crystal structure, as suggested in other research (Mat Yusuf et al., 2020). Based on the height of the peak 2*θ* = 38.00, the crystalline size was calculated with Scherrer’s formula and a value of 5.4 nm was provided.

### Anti-bacterial activities of Mf-AgNPs

#### Agar diffusion assay

**Figure 3:**
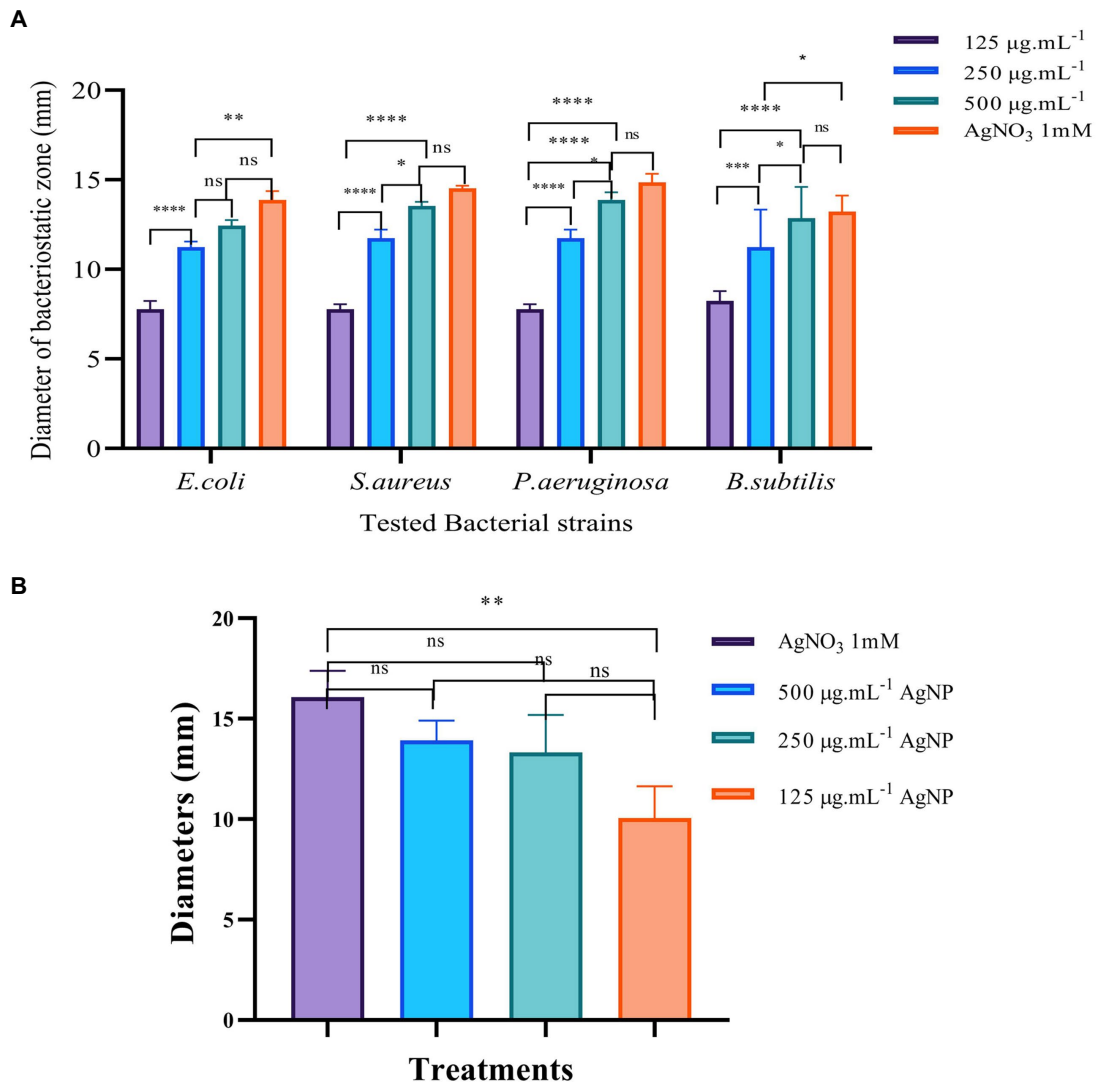
The results of bacteriostatic zone test of Mf-AgNP on four bacterial and *Pectobacterium carotovorum* cells. Panel **(A)** is the results of four model bacteria, and the Panel **(B)** is the results of *P. carotovorum.* Error bars represent standard deviation from the mean of three replicate values (ns non statistically significant difference, *p<0.05, **p<0.01, ***p<0.001, ****p<0.0001).

The bacteriostatic zone experiment is a standard method to study the bacteriostatic ability of substances. In this study, four model bacteria strains including *E. coli*, *S. aureus*, *B. subtilis,* and *P. aeruginosa*, were first selected to investigate the bacteriostatic ability of Mf-AgNPs (Figure 3). The results showed that the plant extract solution alone has not the visible diameter of the bacteriostatic zone, because the bacterial could growth inside of Oxford cup (Figure 3A). In contrast, the Mf-AgNP exhibited significant anti-bacterial activity in a concentration-depending manner (Figure 3A).

Taking *E. coli* as an example, the apparent inhibition zone appeared to be between 125 and 500 μg ml^−1^ Mf-AgNPs. The diameter of the bacteriostatic zone of 125 μg ml^−1^ Mf-AgNP was 7.781 ± 0.469 mm, while the corresponding value was increased to a diameter of 11.234 ± 0.328 mm for 250 μg ml^−1^ Mf-AgNP. In addition, the diameter of 500 μg ml^−1^ Mf-AgNP inhibition zone was 12.349 ± 0.322 mm. The antibacterial zone diameters of 125 and 250 μg ml^−1^ Mf-AgNP were significantly different (*p* < 0.001), and there was no significant difference between 500 μg ml^−1^ Mf-AgNP and 1 mM AgNO_3_ solution. For gram-positive bacterium *S. aureus*, the zone diameters were 7.780 ± 0.285, 11.736 ± 0.483, 13.536 ± 0.231, and 14.452 ± 0.148 mm for 125–500 μg ml^−1^ Mf-AgNP and 1 mM AgNO_3_ solution, respectively. A similar trend was found in this experiment with the other two bacteria, indicating that the Mf-AgNP has a significant inhibitory ability on the growth of both gram-positive and gram-negative bacteria, equivalent to 1 mM AgNO_3_ solution.

For the 2 gram-positive bacteria B.ubtilis and P.aeruginosa, the difference in the inhibitory effect of AgNPs at 250 and 500 μg ml^−1^ did not reach a significant difference. Also for the 2 gram-negative bacteria, the inhibition of AgNP at 250 and 500 μg ml^−1^ achieved only a significant difference (*p* < 0.05). Furthermore, the results of some other studies also revealed that the difference in the antibacterial effect of different concentrations of AgNP was not always significant. Artemisia marschalliana derived AgNPs exhibited similar diameters of inhibition zone from 50 to 100 ppm (Salehi et al., 2016). Thus, we therefoe assumed that in some cases, the antibacterial effects of AgNPs increased in proportion to the concentration of nanosilver solution but were not in a linear fashion.

Here, the concentration of Mf-AgNPs was relatitively higher than the MIC concentration. The same assay was purformed using Mf-AgNPs at concentration from 4 to 16 μg ml^−1^, and the diameters of inhibitory zone ranged from 7.22 ± 0.11 mm to 8.45 ± 0.19 mm, respectively, while the value for 1 mM AgNO_3_ was 13.98 ± 0.28 mm. We also noticed that Rajivgandhi et al. (2019a, 2020a) obtained satisfactory results using the low concentration of AgNPs (5–100 μg ml^−1^) to control *Staphylococcus saprophyticus* or *Staphylococci*. Therefore, we speculated that the differences in the concentration of AgNPs used in different experiments might be related to differences in the antibacterial capacity of AgNPs obtained and the bacterial strains used in various studies.

Next, we analyzed the activity of AgNP against the growth of *P. carotovorum.* The results showed that AgNP also restrained the growth of this bacterium, with the 10.060 ± 1.576 mm bacteriostatic zone for 125 μg ml^−1^ AgNP, 13.317 ± 0.820 mm for 250 μg ml^−1^ Mf-AgNP, 13.939 ± 0.983 mm for 500 μg ml^−1^ AgNP and 16.073 ± 1.325 mm for 1 mM AgNO_3_, respectively (Figure 3B; Supplementary Figure S3). In addition, these results also indicated that the ability to kill *P. carotovorum* was dependent on the concentration of Mf-AgNP, and the ability of 500 μg ml^−1^ Mf-AgNP was comparable to 1 mM AgNO_3_, suggesting the effectiveness of Mf-AgNP.

#### MIC and MBC assay

**Figure 4:**
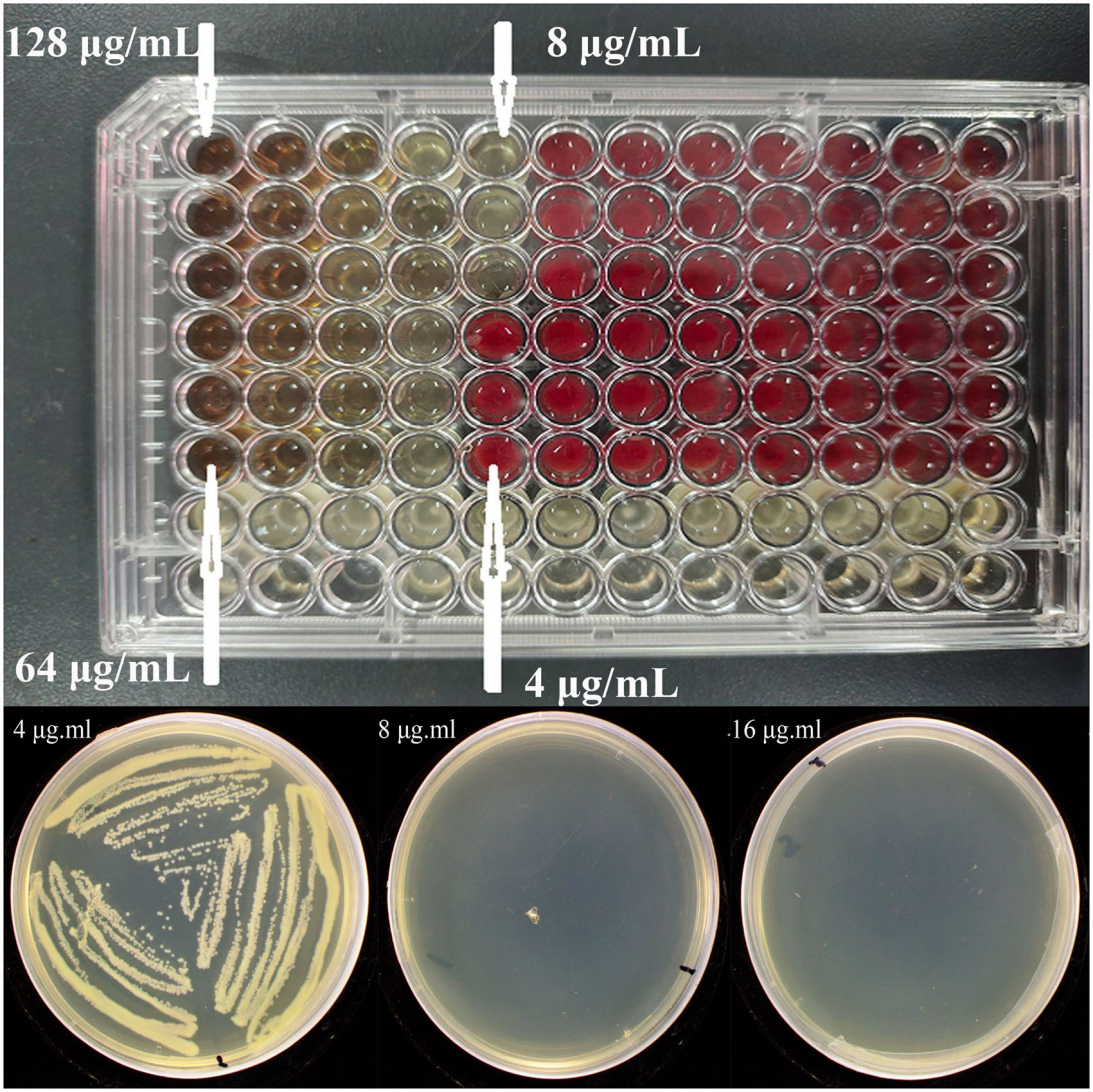
The MIC and MBC results of Mf-AgNP against *P. carotovorum*. The top and bottom images are the MIC and MBC results, respectively.

Based on the results of bacteriostatic zone assay, the synthesized Mf-AgNP obviously showed anti-bacterial activity, hence its function against *P. carotovorum* was further evaluated by MIC and MBC analysis. The test results showed that both the MIC and the MBC value were 8 μg ml^−1^ (Figure 4; Supplementary Figure S4), indicating the high effectiveness in controlling *P. carotovorum*.

#### Growth curve and time killing analysis

**Figure 5:**
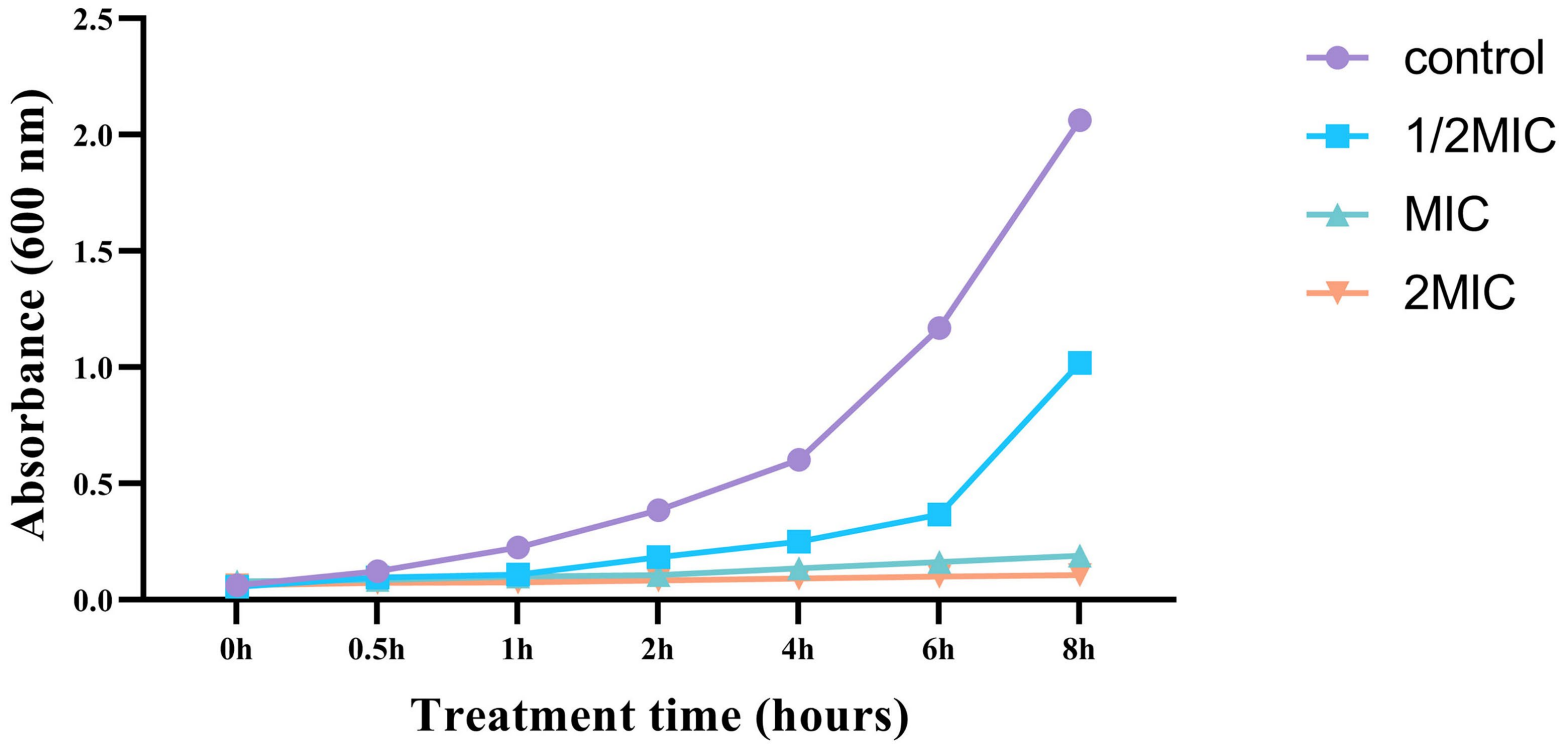
The growth curve of *P. carotovorum* under different concentrations of Mf-AgNP.

The growth curve data demonstrated that different doses of Mf-AgNPs resulted in varying degrees of bacterial growth inhibition (Figure 5). Additionally, the effects of Mf-AgNP stress on cell development were more severe at higher concentrations. For instance, the proliferation of cells treated with 2MIC (16 μg ml^−1^) and 1MIC (8 μg ml^−1^) Mf-AgNP was completely arrested from the start of the assay. After treatment 2 h and thereafter, the difference in OD_600_ values was observed. At this time point, the OD_600_ value of control was 0.387 ± 0.015, obviously higher than that 1/2 MIC (*p* < 0.01), while the absorbance values remained constant for the other two treatments. Up to 6 h of treatments, the OD_600_ values for control and three treatments were 1.170 ± 0.069, 0.367 ± 0.023, 0.164 ± 0.090, and 0.107 ± 0.005, respectively, denoting a significant difference in bacterial growth between all samples (*p* < 0.05 or *p* < 0.01, respectively).

The difference in bacterial growth becomes evident after the fourth hour, and the bacterial growth rate of the control sample is noticeably higher than that of the three AgNP-treated samples. These findings agreed well with those of the MIC and MBC analyses. As a result, this experiment showed that AgNP had a significant inhibitory effect on the growth of *P. carotovorum*.

**Figure 6:**
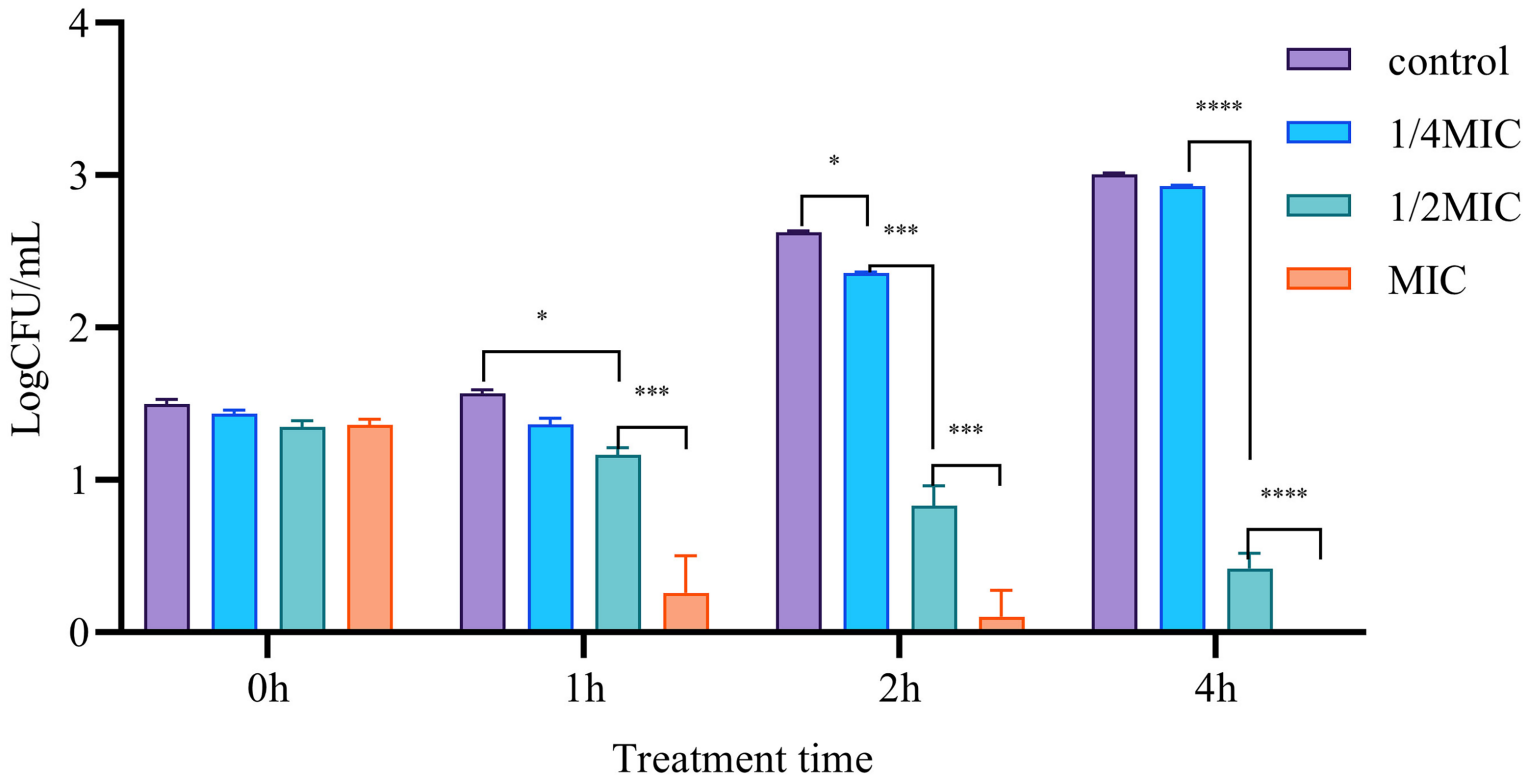
The time-killing results. Error bars represent standard deviation from the mean of three replicate values (ns non statistically significant difference, *p<0.05, **p<0.01, ***p<0.001, ****p<0.0001).

We further performed the Time-killing assay to verify the anti-bacterial function of Mf-AgNP. The results showed that the addition of 4 μg ml^−1^ or 8 μg ml^−1^ Mf-AgNP had remarkable inhibitory effects on bacterial growth, particularly the addition of 8 μg ml^−1^ of AgNP (Figure 6). After 1 h of treatment, 4 μg ml^−1^ of AgNP challenge decreased the Log_CFU_ value to 1.165 ± 0.046, significantly lower than that of 1.568 ± 0.020 of control (*p* < 0.01), while the 8 μg ml^−1^ of AgNP produced a value of 0.259 ± 0.241 (*p* < 0.001), indicating a 25.70% and 84.49% reduction, respectively. Increasing the stress time has been shown to inhibit bacterial cell growth to a greater extent. After treatment for 4 h, the addition of 4 μg ml^−1^ of AgNP showed an inhibitory ratio of 86.19 (0.418/3.006) while 8 μg ml^−1^ of Mf-AgNP completely terminated the growth of cells in plates. In summary, Mf-AgNP exerted an inhibitory effect on bacterial growth, consistent with the conclusions from the growth curve assay.

#### Bacterial cell membrane leakage assay

**Figure 7:**
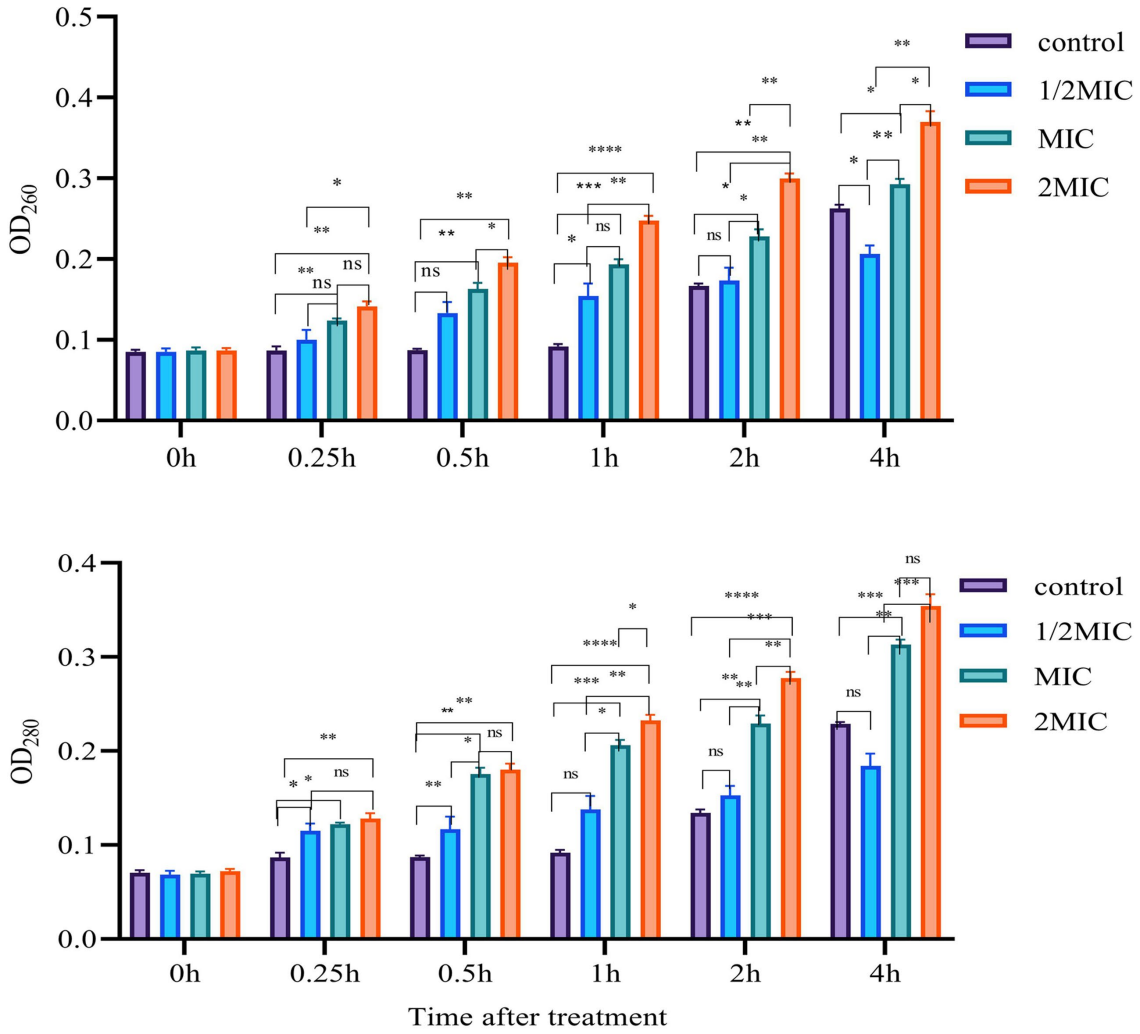
Cell membrane leakage results. Error bars represent standard deviation from the mean of three replicate values (ns non statistically significant difference, *p<0.05, **p<0.01, ***p<0.001, ****p<0.0001).

It is well established that one of the most important antibacterial mechanisms of Mf-AgNP is that it binds to bacterial cell membranes and causes cell membranes to rupture, ultimately leading to the release of the contents (Ibrahim et al., 2020). Here, we measured the absorbance at 260 and 280 nm of the supernatant, which mainly originated from the nucleic acids and proteins released from intracellular components, respectively, to detect the cell membrane leakage under AgNP stress. The results showed that the OD_260_ and OD_280_ values raised following prolonging the treatment time within 0–2 h (Figure 7). After 0.5 h, 4 μg ml^−1^ of Mf-AgNP treatment increased the OD_260_ values to 0.133 ± 0.014, higher than the corresponding value of 0.087 of the control (*p* < 0.05), while the corresponding value of 8 μg ml^−1^ of AgNP treatment was 0.163 ± 0.007. Meanwhile, the OD_280_ value for 8 μg ml^−1^ AgNP treatment 0.5 h was 0.176 ± 0.007, significantly higher than 0.087 ± 0.002 (*p* < 0.01). This difference in absorbance value between control and AgNP treatments was more pronounced after 1 and 2 h exposure. However, there was an exception after stress for 4 h, which was marked by the higher values of OD_260_ and OD_280_ of the control than that of treatment by 4 μg ml^−1^ AgNP. It was hypothesized that this phenomenon was caused by the greater growth of control than that of this treatment.

**Figure 8:**
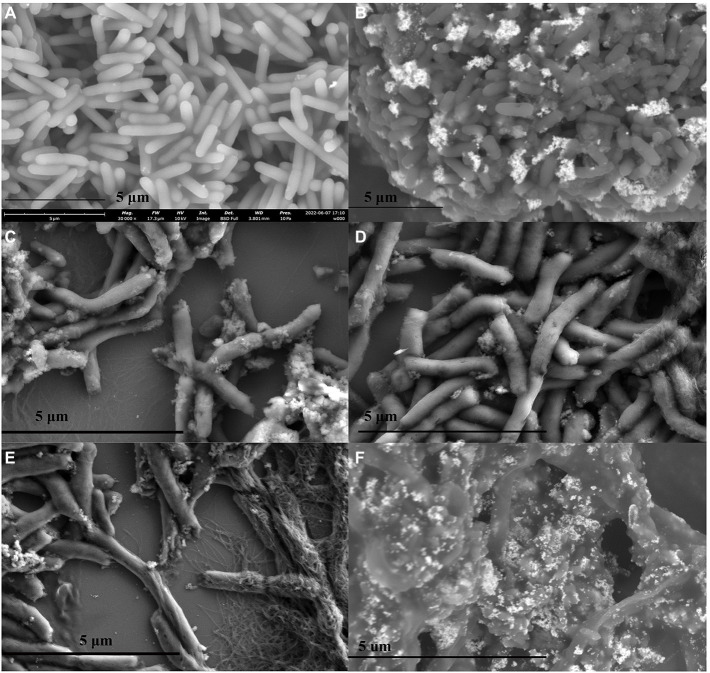
SEM images of cells treated with different concentrations of Mf-AgNP. **(A–F)** denote the images from cells treated with AgNP at 1MIC concentration for 0, 15, 30 min, 1 and 2 h, respectively. The panel **(F)** illustrated several cells that underwent severe structure abnormalities when treated for 2 h.

Besides the absorbance determination assay, we also performed the SEM observation of *P. carotovorum* cells treated with 8 μg ml^−1^ Mf-AgNP at 0, 15, 30, 60, and 120 min time points. As shown in Figures 8A,B, even after 15 min of challenge, the external morphology of the bacterial cells changed significantly, exhibiting a dark color compared to the control. Furthermore, under this treatment, a small number of cells underwent cell surface leakage (Figure 8B), manifested by the appearance of a visible punch on its cell surface. As the treatment time increased to 30 and 60 min, respectively, more cells developed holes and some cells ruptured with losses of membrane integrity (Figures 8C,D), as observes on CuO NP treated ESBL producing bacterial (Rajivgandhi et al., 2019b). After 2 h stress, a large proportion of cells showed structural abnormalities in appearance (Figure 8E), including cell surface breakage, cell distortion, cell fracture, and even cell wall disintegration of some cells, leaving only the cytoskeleton (Figure 8E). Last, bacterial cells with misshapen and fragmentary morphology were observed (Figure 8F), which has been found ESBL positive *P. aeruginosa* cells treated by chitosan/AgNPs (Muthuchamy et al., 2019). The cellular deformation under severe AgNP treatment was also observed by other studies (Ashour et al., 2015; Zada et al., 2018).

#### H_2_O_2_ tolerance

In this study, we used H_2_O_2_ tolerance assay to indirectly evaluate the redox status of bacterial cells under Mf-AgNP treatments at different time points. The results showed that AgNP stress worsened redox status and consequently impaired their tolerance to H_2_O_2_ stress (Supplementary Figure S5)_._ As indicated by the number of colonies formed in H_2_O_2_-containing plates, the results showed that the tolerance of bacteria to H_2_O_2_ decreased significantly with increasing time of Mf-AgNP treatment, particularly on plates containing 0.1 and 0.25 mM H_2_O_2_, while only control bacteria showed weak colony formation on plates containing 0.5 mM H_2_O_2_ and all Mf-AgNP treated bacteria had no colony formation. As shown, the control bacteria at 10-fold gradient dilution grew well on plates containing 0.1 and 0.25 mM H_2_O_2_, while those treated with AgNP at 1/4 MIC concentration for 15 min also grew on these two types of plates, with no significant influence of different dilution gradients on the formation of their colonies (Supplementary Figure S5a–f). However, when the AgNP concentration was increased to 1 MIC, bacterial growth was significantly inhibited and the number of colonies formed was significantly reduced, even on plates containing 0.1 mM of H_2_O_2_ (Supplementary Figure S5c). This decreasing trend was significantly correlated between the increase of the AgNP treatment time and the increase of the H_2_O_2_ concentration, as shown by the fact that there was neither significant colony formation on plates containing 0.25 mM of H_2_O_2_ when treated with 1/2 MIC concentration of Mf-AgNP for 30 min (Supplementary Figure S5d–f), while this trend was more pronounced on plates containing 0.5 mM of H_2_O_2_.

### Suppression of biofilm formation

**Figure 9:**
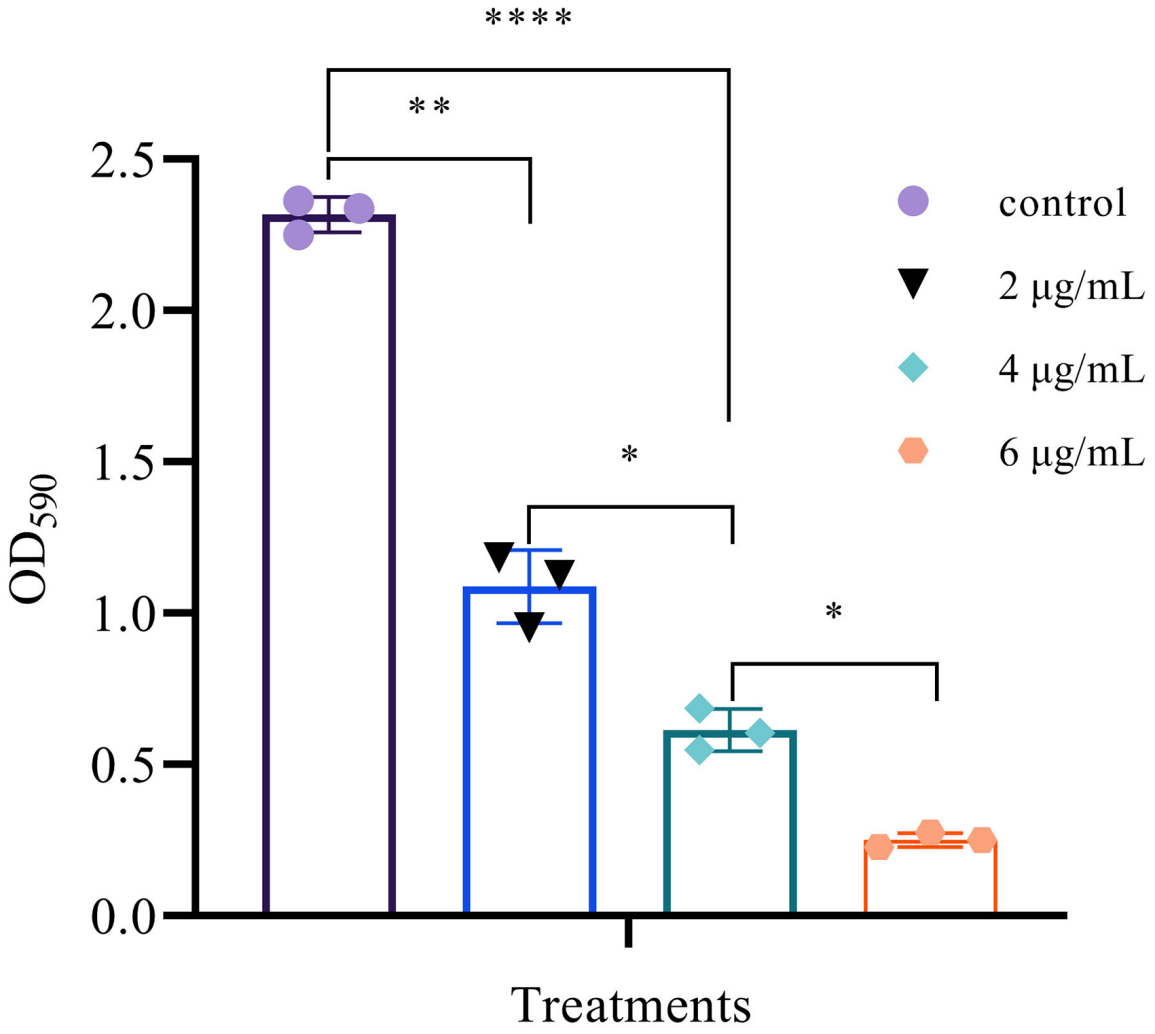
Suppression of biofilm formation of different concentrations of AgNPs. Error bars represent standard deviation from the mean of three replicate values (ns non statistically significant difference, *p<0.05, **p<0.01, ****p<0.0001).

The formation of biofilm is a very complex process that provides bacterial cells with a relatively stable environment in which to survive and proliferate. Therefore, preventing this process would thus benefit the antibacterial function (Hussain et al., 2019). As shown in Figure 9, different concentrations of Mf-AgNP can inhibit biofilm formation to varying degrees in *P. carotovorum.* When 6 μg ml^−1^ Mf-AgNP was used, bacterial biofilm formation showed a significant decrease compared to the control (*p* < 0.01), and the amount of bacterial biofilm formation decreased significantly as AgNP concentration increased. The value of OD_570_ absorbance was 0.252 ± 0.023 after 6 μg ml^−1^ Mf-AgNP treatment, decreasing to 10.87% of control (2.318 ± 0.059), and the corresponding values for those treated with 4 and 2 μg ml^−1^ AgNP were 0.614 ± 0.072 and 1.088 ± 0.121, respectively, decreasing to 26.49% and 46.94% of the control. When using another calculation method, the bacterial film destruction rate was 89.13% for the 6 μg ml^−1^ AgNP treatment, while the 4 and 2 μg ml^−1^ AgNP treatments were 73.51% and 53.04%, respectively (Habibipour et al., 2019). Therefore, the Mf-AgNPs can remarkably inhibit the establishment of the biofilm of *P. carotovorum* and then exert an inhibitory effect on the reproduction of *P. carotovorum*.

### Inhibition of enzymatic activities

**Figure 10:**
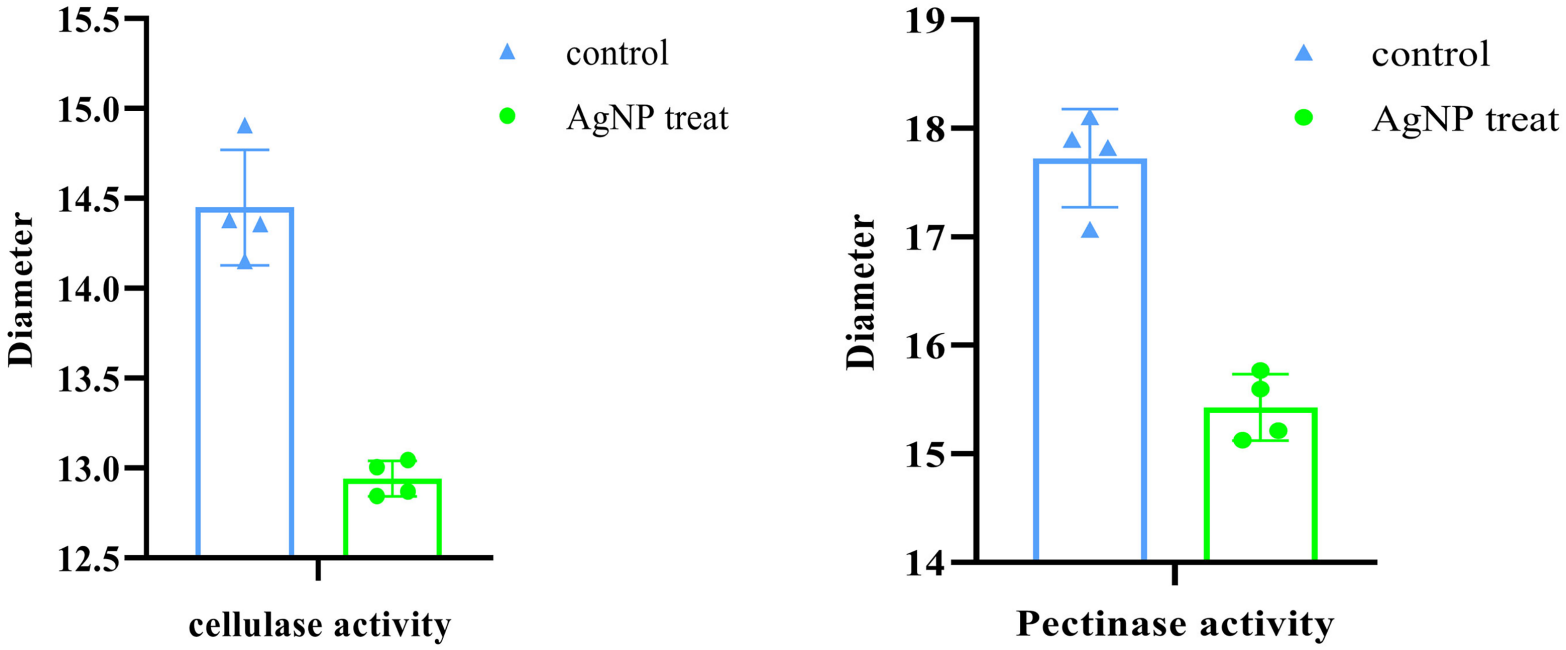
Inhibition of extracellular pectinase and cellulase activities.

The synergistic degradation of plant cell walls caused by the bacterial enzymes pectinase and cellulase that they release is a critical step in the pathogenesis of *P. carotovorum* (Cui et al., 2019). In addition to accelerating bacterial cell death, blocking the actions of these two extracellular enzymes aided in the prevention of infection in the cabbage. To avoid interference from the significant growth suppression caused by AgNPs at 4 μg ml^−1^ or higher concentrations, we treated bacteria with 2 μg ml^−1^ Mf-AgNP before analyzing the activities using the plate method (Figure 4). The findings demonstrated that these two extracellular enzymes’ activities might be visibly reduced by such a low quantity of Mf-AgNPs (Figure 10). Pectinase’s transparent zone diameter reduced from 17.72 ± 0.45 mm of the control to 15.43 ± 0.31 mm (*p*<0.05), whereas cellulase’s transparent zone diameter decreased from 14.45 ± 0.32 mm to 12.95 ± 0.10 mm (*p*<0.05). These findings unequivocally established Mf-AgNP’s enzymatic inhibitory action on enzymes crucial to *P. carotovorum* pathogenesis.

### Synergistic inhibition of AgNP and Zhongshengmycin

The results showed that AgNP had a significant synergistic effect with Zhongshengmycin. In this study, the MIC of AgNP alone against *P. carotovorum* was 8 μg ml^−1^, while the MIC of 3% zhongshengmycin alone was 50 μg ml^−1^ (Supplementary Figure S4). As demonstrated by the checkerboard assay, the Mf-AgNP had the potent antibacterial ability when combined with zhongshengmycin, with apparent synergistic effects (Supplementary Figure S5). In this regard, it was found that the MIC of both substances was significantly reduced, to 1 μg ml^−1^ for AgNP and 3.063 μg ml^−1^ for zhongshengmycin (Supplementary Figure S5 F7 well), respectively, indicating that these two substances have a synergistic effect achieved to reduce the use of antibiotics while also playing a role in environmental protection and reducing the production of drug-resistant bacteria (Rai et al., 2012; Liao et al., 2019; Qais et al., 2019).

The outcomes demonstrated that Zhongshengmycin and Mf-AgNP had a sizable synergistic impact. In this investigation, the MIC of 3% zhongshengmycin alone was 50 μg ml^−1^, while the MIC of Mf-AgNP alone was 8 μg ml^−1^ for *P. carotovorum*. The checkerboard assay results clearly demonstrated that Mf-AgNP combined with zhongshengmycin has strong antibacterial properties, with evident synergistic effects. In this regard, it was discovered that the MIC of both substances was significantly lowered, to 1 μg ml^−1^ for AgNP and 3.063 μg ml^−1^ for zhongshengmycin (Supplementary Figure S5 F7 well), respectively, indicating that these two substances have a synergistic effect achieved to reduce the use of antibiotics while contributing to environmental protection and reducing the production of drug-resistant bacteria (Rai et al., 2012; Liao et al., 2019; Qais et al., 2019).

Next, the synergistic inhibitory ability of Mf-AgNP and zhongshengmycin was quantitatively examined by calculating their FIC index. As a result, the FIC = 1/8 + 3.0625/50 = 0.174. It is generally accepted that the FIC of two substances below 0.5 indicates a significant synergistic effect between them for bactericidal activity (Loo et al., 2018). Therefore, the inhibition of *P. carotovorum* by AgNP and zhongshengmycin is highly synergistic when used in combination, which can provide a reference for the development of new drugs to control the cabbage soft rot disease.

**Figure 11:**
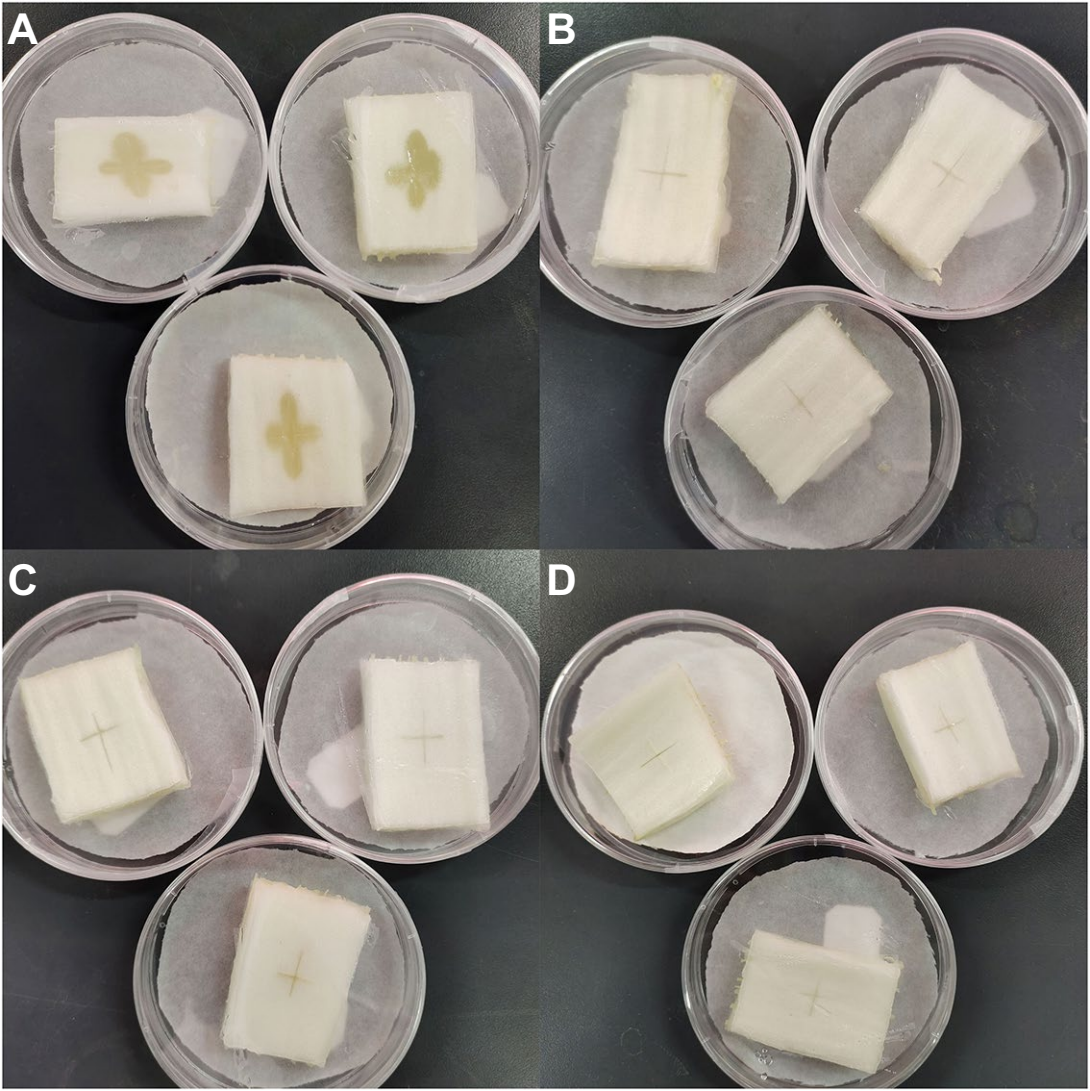
Results of *in vitro* experiments on the inhibitory effect of AgNPs in combination with zhongshengmycin on *P. carotovorum*. The panel **(A)** is the result of the control without being treated with AgNP or zhongshengmycin, while **B–D** are the results of explants treated with 10 μg ml^−1^ AgNP, 60 μg ml^−1^ zhongshengmycin, and the combined of 2 μg ml^−1^ + 5 μg ml^−1^ zhongshengmycin.

Finally, we conducted an *in vitro* experiment using cabbage explants to confirm the synergistic inhibitory effect of Mf-AgNP and zhongshengmycin on *P. carotovorum* infection (Figure 11). As a negative control, the explants injected with *P. carotovorum* alone exhibited obvious growth of bacteria and soft rot. In contrast, the cabbage petioles treated with 10 μg ml^−1^ MF-AgNP or 60 μg ml^−1^ zhongshengmycin showed no bacterial growth and the development of soft rot. The reason for using Mf-AgNP and zhongshengmycin slightly higher than their MIC concentration was to avoid dilution in explants since the volume of the explants was significantly larger than the wells in a 96-well plate. Referenced the result of FIC calculation formula, we combinationly applied 2 μg ml^−1^ MF-AgNP and 5 μg ml^−1^ zhongshengmycin and found a complete arresting of bacteria growth on the explants after 12 h infection, indicating that the combination of the two can significantly reduce the demand of antibiotics used in the control of soft rot.

### Assessment of *in vivo* toxicity

**Figure 12:**
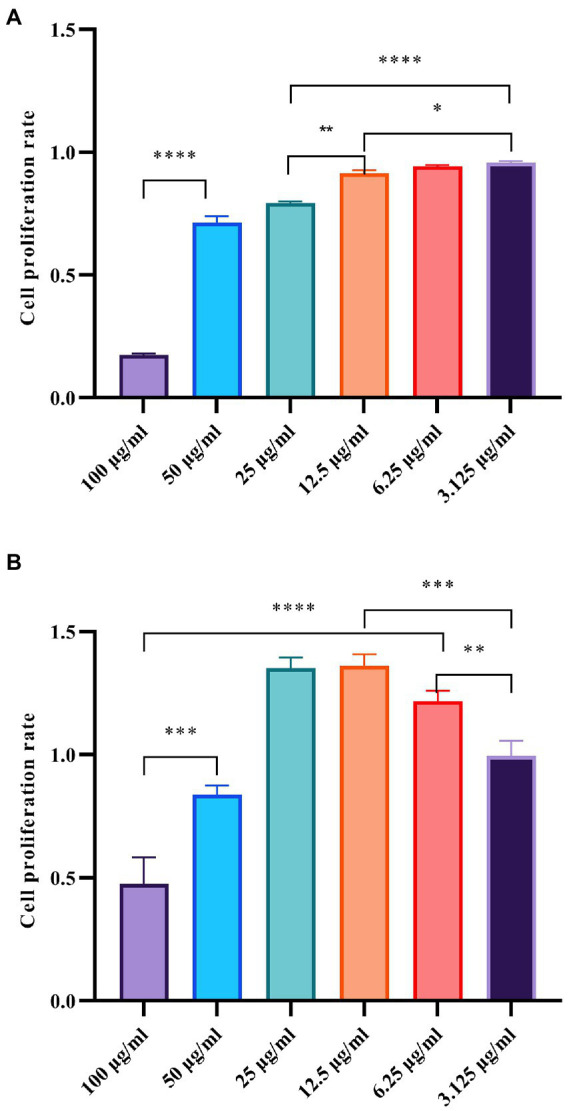
Cytotoxicity of AgNPs on CFPAC1 and HFS cells was determined by MTT assay. The panels **(A, B)** refer to the cell proliferation ratio of CFPAC1 and HFS cells under AgNP treatment for 24 h, respectively.

An important aspect affecting the application of AgNP is its safety for consumption, especially in terms of cytotoxicity or biocompatibility with human cells. In this study, two different human cell lines were used to analyze the *in vivo* toxicity of the Mf-AgNPs. The results showed that Mf-AgNPs had moderate toxicity for pancreatic cancer cell lines, while being safer for human skin fibroblast cells (Figure 12). In this study, the inhibitory effect of Mf-AgNP on CFPAC1 cell proliferation was dependent on its concentration. Under stress with 3.125 μg ml^−1^ AgNP, the cell proliferation ratio was 0.958 ± 0.006, significantly higher than that under 6.25 and 12.5 μg ml^−1^ AgNP (*p* < 0.05). When AgNP concentration increased to 25 μg ml^−1^ or higher, the corresponding ratio decreased to 0.793 ± 0.009 and finally to 0.174 ± 0.007. In contrast to CFPAC1 cells, the HFS cells were insensitive to the challenge of Mf-AgNPs, especially at low concentrations. At AgNP concentration at 25 μg ml^−1^ or less, the promoting effects on cell viability were observed, with the maximum value being 1.362 ± 0.047 under 12.5 μg ml^−1^ treatment. On the other hand, using AgNP at 50 and 100 μg ml^−1^ resulted in a decrease in cell viability ratio to 0.837 ± 0.038 and 0.475 ± 0.107, respectively.

For the human pancreatic cancer cell line, the cytotoxicity of AgNP was not yet present. Therefore, we compared the effects of Mf-AgNP on other human cell lines. In the results of Rajivgandhi et al. (2019b), the bio-synthesized CuO NP at MIC concentration exhibited 2.5% inhibition against human RBC. For human hepatoma cell line HepG2, the AgNP synthesized from *Carduus crispus* showed a slight decrease in cell viability after 24 h of treatment (Urnukhsaikhan et al., 2021). Considering that human skin cells have the most direct contact with AgNP materials, the results of this study indicate that Mf-AgNP has high biosafety. Collectively, these results showed that the Mf-AgNP was a compound with high biocompatibility to human skin fibroblast cells, as well as a promising drug for the treatment of pancreatic cancer.

In conclusion, Mf-AgNPs with typical physicochemical properties of green synthesized Mf-AgNPs demonstrated significant inhibitory effects on the important plant pathogen *P. carotovorum*, as evidenced by lower MIC and MBC values, impediment bacterial growth, induction of collapse of bacterial cell structure and leakage of contents, inhibition of extracellular pectinase and cellulase activities, impairment of biofilm formation and disruption of the dynamic balance of cellular ROS metabolism. More importantly, Mf-AgNP synergized significantly with zhongshengmycin, lowering the concentrations of Mf-AgNP and zhongshengmycin used on cabbage explants to control *P. carotovorum* infection. To the best of our knowledge, this is the first report on the application of plant-mediated AgNP to inhibition of the Chinese cabbage bacterial pathogen. The findings of this study shed light on how to control Chinese cabbage soft rot and provided a new idea for reducing antibiotics consumption in vegetable crop production.

## Discussion

In general, the shape and size characteristics of AgNPs are the determining factors affecting their biological functions, particularly their antibacterial ability and biocompatibility, which consequently determines their application potential (Agnihotri et al., 2014). For green synthesis of AgNP, these properties were strongly influenced by the capacity and the composition of the reducing substances in the plant extracts. The obtained Mf-AgNPs have desirable properties for antibacterial purposes, such as a uniform morphology and a narrow particle size distribution. In this regard, these monodisperse AgNPs have a near-spherical shape, an average size of 13.19 nm in diameter, and a zeta-potential of −11.5 mV. Due to these physicochemical characteristics, Mf-AgNPs have a large specific surface area, are moderately stable, highly cytotoxic to bacterial cells, and are biologically safe to human cells.

In this study, the *M. fortunei* extract solution showed no antibacterial functions, and several factors affecting its antibacterial capacity were addressed. Fitrst, the plant extracts have not been concentrated or lyophilized, resulting in an decreasing in effective concentration and antibacterial functions such as berberine. Second, in many cases, antibacterial plant components were mainly dissolved in organic solvents such as ethanol and methanol, while aqueous extracts showed lower antibacterial capacity (Yuan et al., 2017a).

The antibacterial performance of spherical AgNP is usually acknowledged to be lower than that of triangular AgNP, while biosafety is higher than that of triangular AgNP. Meanwhile, the smaller the particle size, the greater AgNP’s antibacterial power, but the lower its biosecurity. As a result, the AgNP produced in this investigation had decreased MIC and MBC values and demonstrated improved antibacterial activity. Furthermore, Mf-AgNP (100 μg ml^−1^) cytotoxicity was modest against human skin fibroblasts but high against pancreatic cancer cells, with a reduction of cell growth rates of 15.03% and 82.88%, respectively. These data revealed that the AgNP produced in this study has strong antibacterial properties as well as low cytotoxicity and that it has practical application potential. Metallic nanomaterial biosafety has been a major concern, and it is intimately related to the type of cells utilized, as well as the physicochemical features of the nanomaterials themselves. The toxicity of Mf-AgNP on human cells was investigated using an MTT-based approach for the proliferation of two cell types in this work and their biosaftity to human HSF cell was revealed (Figure 12). However, a more in-depth and thorough evaluation of their toxicity utilizing other systems such as human RBC and the model animal *Artemia franciscana*, is required. Particularly, the *Artemia* nauplii martality is sensitive to metal NPs and easy to handle, providing powerful tool for evaluate the toxicity of NPs (Muthuchamy et al., 2019; Rajivgandhi et al., 2019c).

Although the mechanisms underlying the antibacterial ability of AgNP are still not fully elucidated, several mechanisms have been addressed, mainly including induction of cell membrane damage, influencing of a variety of biomolecular activities, and stimulation of ROS production. In this study, the antibacterial function of Mf-AgNP was analyzed using several biochemical experiments, and the results showed that Mf-AgNP had multiple antibacterial mechanisms.

First, the optical absorption results of bacterial supernatant after AgNP treatment indicated that AgNP-induced disruption of cell membrane integrity and leakage of cell contents were present, and the severity of leakage was influenced by AgNP concentration and stress time (Figure 7). Furthermore, the result regarding cell membrane rupture and subsequent severe cellular structural destruction. Was supported by the direct observations in the SEM images (Figure 8).

Second, the results of H_2_O_2_ tolerance of bacterial cells showed that treatment with Mf-AgNP at a concentration of 0.5 × MIC or higher for 0.25 h on 0.25 mM H_2_O_2_-containing plate showed severely inhibited cell growth and colony formation (Supplementary Figure S5c). The concentration of AgNPs used and the duration of treatment had a significant and positive correlation with the detrimental effect on bacterial growth. Although we were unable to detect ROS production under Mf-AgNP treatment for device restriction, we hypothesized that these findings were related to the increased generation of ROS and the dysfunction of the cellular anti-reactive oxygen species system under AgNP stress. As a result of the accumulated ROS, bacteria produced a series of abnormal cell functions, eventually leading to cell death (Kim et al., 2019).

Third, the biofilm is a community of bacteria surrounded by mainly extracellular polymeric substance (EPS), which provides a protective environment for bacterial growth, while decreasing the effectiveness of antimicrobial substances and protecting bacteria from immune system effects. Simultaneously, the presence of biofilm lays the groundwork for the development of bacterial drug-resistance (Hall-Stoodley et al., 2004). With a dose-depend manner, Mf-AgNP inhibited biofilm formation in *P. carotovorum* to varying degrees, from 89.13% for the 6 μg ml^−1^ AgNP to 53.04% for 2 μg ml^−1^AgNP. Hussain et al. (2019) discovered that AgNPs at a concentration lower than the MIC could effectively reduce biofilm formation. As a result, the inhibition of biofilm formation induced by Mf-AgNP may be a key mechanism contributing largely to their antibacterial roles against *P. carotovorum*. However, the detailed mechanism of Mf-AgNPs impairing biofilm formation needs to be explored further, as previous research has shown that AgNPs can reduce EPS production, demolish biofilm structure, and suppress the Quorum sensing (QS) process (Hussain et al., 2019; Rajivgandhi et al., 2022b, 2022c).

Fourth, we investigated the inhibitory effect of AgNP at low concentrations on the activities of the two main *P. carotovorum* extracellular infection-associated enzymes and found that while colony growth on plates was unaffected, the activities of pectinase and cellulase were significantly inhibited (*p*<0.05). These findings made it abundantly evident that Mf-AgNP can prevent bacterial growth and survival *via* a variety of methods. It gives Mf-AgNP a robust antibacterial activity and lessens the development of bacterial drug resistance mechanisms, making Mf-AgNP useful in practical applications.

Currently, the extensive use of antibiotics to prevent and control of detrimental effects of pathogenic bacteria on crop growth not only compromises food safety problems due to antibiotic residues in agricultural products, but it also encourages the development of resistance mechanisms in pathogenic bacteria, which exacerbates the issue of antibiotic abuse (Byrne et al., 2019). In many studies, the combined use of green synthetic AgNP and many different antibiotics showed synergistic properties against pathogenic microorganisms (Panáček et al., 2015; Aabed and Mohammed, 2021). In the present study, the co-use of Mf-AgNP and zhongshengmycin showed a very significant synergistic effect, and the MIC values of both against *P. carotovorum* were reduced to a tenth and 6% of the original values, respectively, with an FIC index of 0.174. Experiments using cabbage explants showed that the combinational using use of 2 μg ml^−1^ Mf-AgNP and 5 μg ml^−1^zhongshengmycin under the conditions of wholly inhibited the infestation of *P. carotovorum* on cabbage tissues. In summary, these results indicated that the Mf-AgNP obtained in this study is able to reduce the use of antibiotics significantly and may become a new antimicrobial substance that is safe and efficient, thus helping to improve the safety of agricultural products for consumption and protect human health.

## Data availability statement

The original contributions presented in the study are included in the article/Supplementary material, further inquiries can be directed to the corresponding author.

## Author contributions

ZW planned the research. SX and HJ performed the research. ZW and HZ analyzed the data. ZW and SX wrote the manuscript with contributions from HJ and HZ. All authors contributed to the article and approved the submitted version.

## Conflict of interest

The authors declare that the research was conducted in the absence of any commercial or financial relationships that could be construed as a potential conflict of interest.

## Publisher’s note

All claims expressed in this article are solely those of the authors and do not necessarily represent those of their affiliated organizations, or those of the publisher, the editors and the reviewers. Any product that may be evaluated in this article, or claim that may be made by its manufacturer, is not guaranteed or endorsed by the publisher.
