# Supplementary material for: Green synthesis of silver nanoparticles from Mahonia fortunei extracts and characterization of its inhibitory effect on Chinese cabbage soft rot pathogen
Source: Front Microbiol. 2022 Oct 21;13:1030261. doi: 10.3389/fmicb.2022.1030261 (PMC9635054; doi:10.3389/fmicb.2022.1030261)
Supplement: Supplementary file 1 [file Data_Sheet_1.docx]

Supplementary Material

## Supplementary Table

## **Supplementary Table1.** The peaks assignments of FTIR spectra

| **Peak position (cm^-1^)** | **Peak assignments** | **Vibrational mode** | **References^a^** |
| --- | --- | --- | --- |
| 3417 | –OH of phenols and carboxylic acids | stretching | Yang et al., 2021; Ashour et al., 2015 |
| 2924 | C–H bond of aliphatic acids or aliphatic hydrocarbons | asymmetric stretching | Yang et al., 2021; Ashour et al., 2015 |
| 1604 | N-H of peptide | stretching | Doi:10.3390/app10175918 |
| 1384 | O–C–H and C–O–H of carbohydrates or hydroxyl flavones | bending | Ibrahim et al., 2020; Yusuf et al., 2020 |
| 1076 | C-O (carboxylic acid) | stretching | Saratale et al., 2017 |
| 613 | C-H of aliphatic and aromatic compounds | bending | Reddy et al., 2021 |

^a^ All indicated publications are in the reference list except for the article that identified with its doi number

## Supplementary Figures


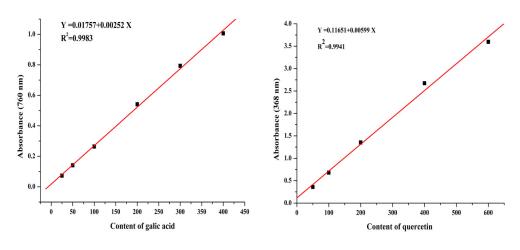


**Supplementary Figure 1.** The standard curve for TPC and TFC content determination

The left panel refers to the curve for TPC content determination, while the right one for TFC, respectively. The OD_760_ values of extract solution at concentration of 5g/L^-1^ was 0.906±0.027, and the OD_368_ values was 1.562±0.064, respectively.


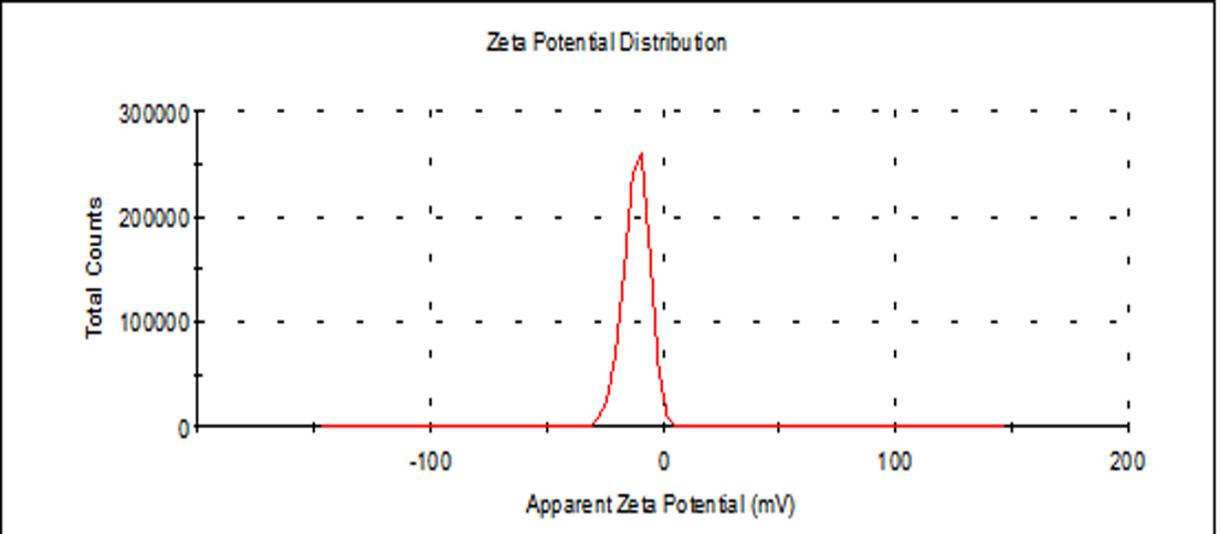


**Supplementary Figure 2.** The zeta-potential curve of Mf-AgNP


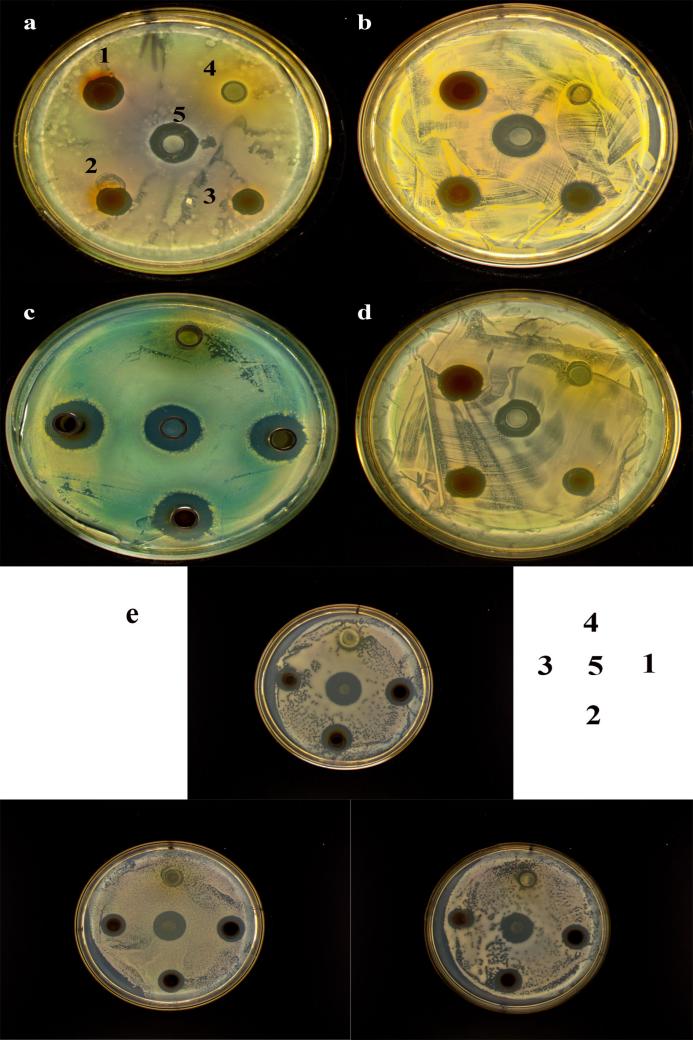


**Supplementary Figure 3.** The images of bacteriostatic zone test of Mf-AgNP on four bacterial and *P.carotovorum* cells

^a^ *E.coli*, ^b^ *S.aureus*, ^c^*P.aeruginosa,* ^d^ *B.subtilis,* ^e^ *P.carotovorum.* The numbers 1 to 5 refer to the zones treatd by 500 μg.mL^-1^, 250 μg.mL^-1^ , 125 μg.mL^-1^ , extract and 1 mM AgNO_3_, respectively.


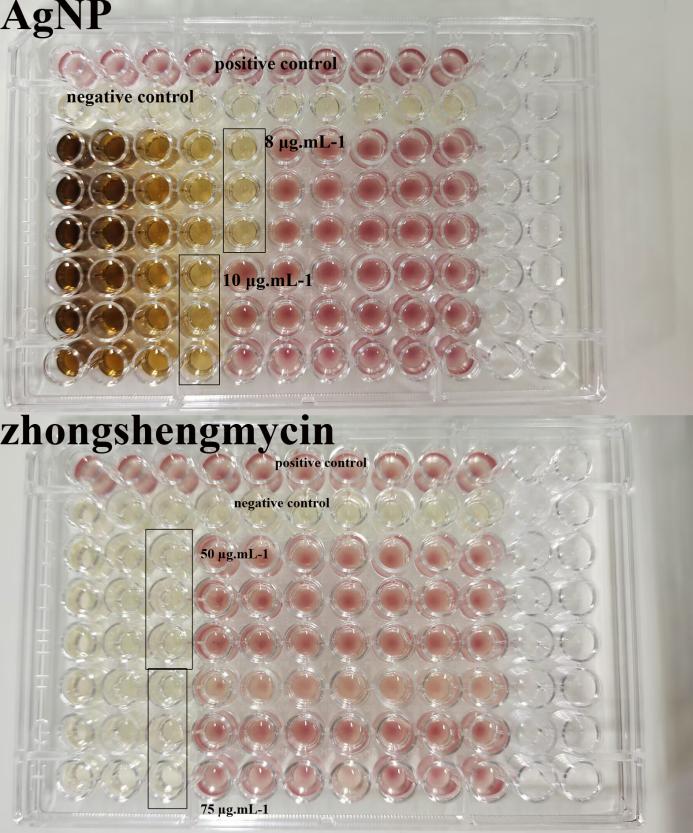


**Supplementary Figure 4.** The MIC results of Mf-AgNP and Zhongshengmycin

The wells E3-E5 were added AgNP to 8 g.mL^-1^ for the MIC result of Mf-AgNP, while the wells A3-A5 were 256 g.mL^-1^. In addition, AgNP at a concentration of 80 g.mL^-1^ was added to wells A6-A9.

The concentrations of zhongshengmycin used in the experiments were 200 g.mL-1 in wells A3-A5, and 300 g.mL^-1^ in wells A6-A9.


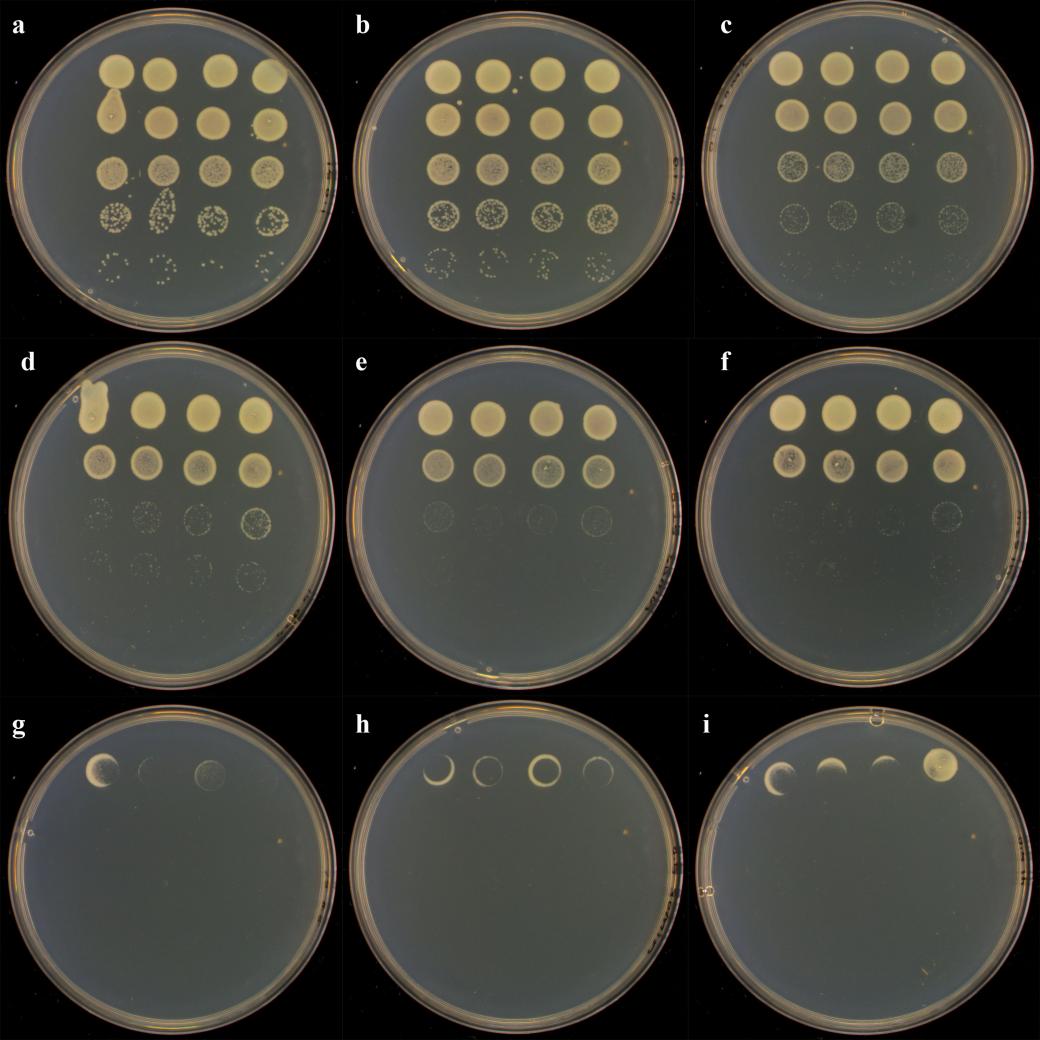


**Supplementary Figure 5.** The H_2_O_2_ tolerance assay results

The symbols a to c refer to the plates containing 0.1 mM H_2_O_2,_ while d to f to 0.25 mM H_2_O_2,_ and g to i to 0.5 mM H_2_O_2_. On each plate, from left to right are four clones formed by 10-fold gradient dilution of bacteria, while from top to bottom, are the control, 1/4 MIC concentration, 1/2 MIC concentration, 1 MIC concentration and 2 MIC concentration treated bacterial solutions, respectively. a to c, d to f and e to i are the AgNP treated bacterial solutions for 15 min, 30 min and 1 hour, respectively.


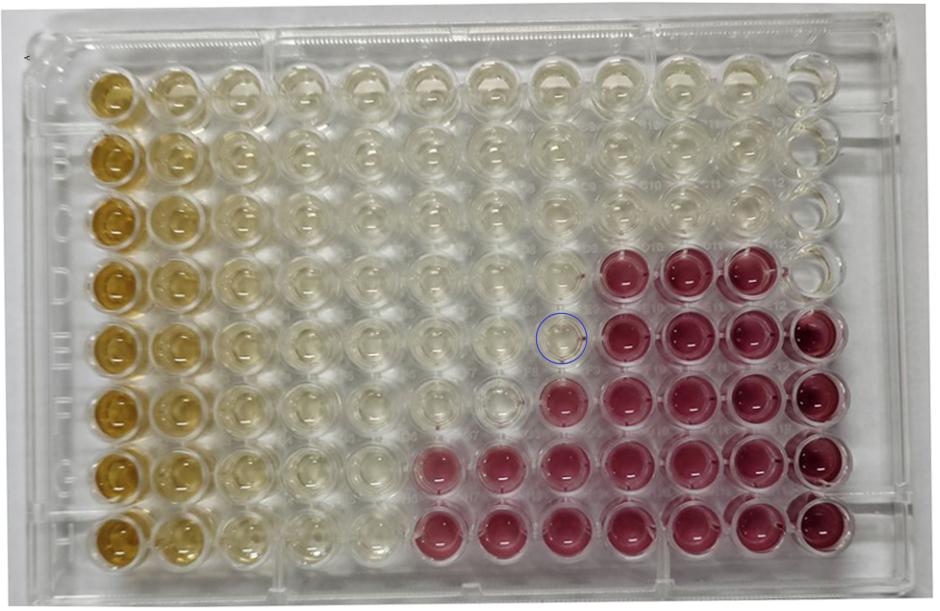


**Supplementary Figure 6.** The synergistic effect of AgNP and zhongshengmycin on inhibition the growth of *P. carotovorum*

This assay was conducted using checkerboard methods. The concentration range of zhongshengmycin was 200, 100, 50, 25, 12.5, 6.25 and 3.1 μg.mL^-1^ with 7 gradients (from the row A to row F), while the concentration of AgNP was set to 200, 100, 50, 25, 12.5, 6.25, 3.12, 1.6, 0.8, 0.4, 0.2 and 0.1 μg.mL^-1^ with 12 gradient sets (from the 1^st^ column to 12^th^ column).
